# An RNA Language Model trained on sequence alone reveals the structural logic of Internal Ribosome Entry Sites

**DOI:** 10.64898/2026.05.19.726202

**Published:** 2026-05-20

**Authors:** Adam Sychla, Pierre Bongrand, Grant Yang, Jacob Rulison, R. Alexander Wesselhoeft, Namita Bisaria, Silvi Rouskin

**Affiliations:** 1 Department of Microbiology, Harvard Medical School, Boston & 02115, USA.; 2 Kenneth C. Griffin Graduate School of Arts and Sciences, Harvard University, Cambridge & 02138, USA.; 3 Gene and Cell Therapy Institute, Mass General Brigham, Cambridge & 02139 USA.; 4 AIRNA Corporation, Cambridge & 02139 USA.

## Abstract

Viral RNA genomes are among the most information-dense codes in biology. In picornaviruses, translation depends entirely on Internal Ribosome Entry Sites (IRESes), yet their structures remain largely unresolved. Previous studies either screened short IRES fragments in high throughput or characterized full-length elements individually. Here, we profile 96 full-length IRESes across six cell types, revealing that recently described Type V IRESes double the activity of EMCV, the standard in bioengineering, and that most IRESes exhibit significant tissue tropism. We introduce Albatross, an RNA language model fine-tuned on 50,000 IRES sequences. Trained on sequence alone, Albatross predicts IRES structures with precision comparable to chemical probing, outperforming covariation analysis. We generate structure maps for ~75,000 full-length IRESes and show that structural discovery scales with model size.

Picornaviruses (*Picornaviridae*) are a diverse and widespread family of RNA viruses with historic, ongoing, and emergent global health impacts, particularly among pediatric populations (1–7). Members of this ubiquitous viral family are capable of infecting a wide variety of tissues, including neural, hepatic, cardiac, gastrointestinal, and respiratory, with outcomes ranging from asymptomatic infections to permanently debilitating diseases (8–12). Beyond causing human diseases including poliomyelitis, the common cold, and hepatitis A, picornaviruses infect nearly all known vertebrates, posing a substantial risk of zoonotic spillover (13).

Picornaviruses typically encode a single polyprotein from their small (~8 kb), positive-sense RNA genomes, with cap-independent translation initiation driven by large (>300 nt) structured RNA elements called Internal Ribosome Entry Sites (IRESes) (8). These structures are conventionally classified into five broad structural types (I-V). The first types were identified in the late 1980s in poliovirus (Type I) and encephalomyocarditis virus (EMCV) (Type II), while the most recent, Type V, was described in 2011 in several genera including kobuviruses (14–18).

IRESes are well known to exhibit tissue and host-specific activities (9,19). Such tropism impacts a broad range of emergent behaviors including spillover risk and severity of disease, making them a crucial target for antiviral therapies and pre-pandemic surveillance. Beyond their biological significance, IRESes have found broad utility in biotechnology, with the EMCV IRES being a standard tool in bioengineering and gene therapy (20–22).

Although individual IRESes have been extensively studied, family-wide rules governing IRES function and tropism remain poorly understood. While massively parallel DNA synthesis enables high-throughput studies of short (~200 nt) IRES fragments, it cannot yet produce at scale the full-length sequences (400–1500 nt) needed to capture complete IRES structures. As a result, our understanding of full-length IRES architecture and evolution is largely limited to individual examples, and most IRES structures remain experimentally unresolved.

Thermodynamics-based RNA folding algorithms generally perform well on short sequences but often require experimentally-derived constraints for longer sequences such as IRESes (23, 24). Such models also become computationally intractable when accounting for many unknown nested, higher-order, or long-range interactions (25, 26). Meanwhile, predictive methods using machine learning have suffered from limited availability of high-quality RNA structure training data. Many models perform well on shorter sequences or in specific contexts but quickly fail when asked to extrapolate into new RNA families (27–30).

Covariation analysis remains one of the best approaches for determining high-confidence RNA secondary structures (31, 32). However, these approaches depend on multiple sequence alignments of several closely related sequences with mutated and compensated base pairs to inform the analysis, which are often lacking, especially for highly divergent RNA elements.

For this work, we sought to find broad, family-wide patterns in IRES activity and structure. Because precise IRES boundaries are often ill-defined, especially for divergent or understudied viruses, we generally use the term IRES throughout to refer to full picornaviral 5′ UTRs, while acknowledging that not all sequences within these regions necessarily contribute to translation initiation. We measured translation from 96 full-length IRESes across six cell types, creating a comprehensive IRES functional atlas, and generated family-wide structure maps using chemical probing. Our results revealed that the recently described Type V IRESes exhibit double the translational activity of the widely used EMCV IRES, and that the majority of IRESes exhibit significant tissue tropism.

To address the structural bottleneck, we developed Albatross, an RNA Language Model fine-tuned from RiNALMo on ~50,000 IRES sequences using only RNA sequence data and a masked-token prediction objective (33). Despite receiving no external knowledge of secondary structure, base pairing rules, or thermodynamics, the model learned an encoding of IRES structure from sequence alone. Using a dependency mapping framework, Albatross recapitulated experimental RNA secondary structures of 96 IRESes with precision comparable to chemical probing, outperforming covariation analysis, and generated high-quality structure maps for ~75,000 more (34). Systematic comparison across model sizes indicates that scaling alone will unlock deeper structural predictions, pointing to a clear path forward for RNA structure discovery.

## Generating a cap-free circRNA library of full-length IRESes

**Figure 1:**
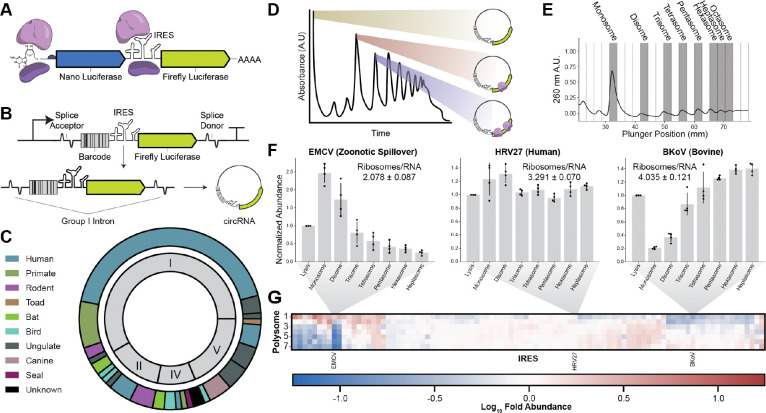
A circRNA system enables multiplexed IRES activity measurement. (**A**) Bicistronic dual reporter assay. (**B**) circRNA design with barcoded full-length IRESes circularized via Group I Intron ribozyme. (**C**) Library composition by host (outer) and IRES type (inner). (**D,E**) Polysome fractionation trace and resolved fractions. (**F**) Representative polysome distributions for EMCV, HRV27, and BKoV. (**G**) Polysome heatmap for all 96 IRESes. All data from HEK293T cells.

IRES activity is traditionally screened for using dual reporter assays (Figure 1A). However, these assays are confounded by multiple sources of false positives. The test sequence may harbor cryptic promoters or splice sites that generate monocistronic transcripts of the downstream reporter (35). Even in the absence of these artifacts, translational readthrough can range from 0.1 to 1.5% and reach as high as 35% depending on sequence context, and such assays cannot distinguish ribosomal reinitiation from *bona fide* IRES activity (35–38). To overcome these challenges, we turned to an *in vitro* generated circRNA system that enables high-throughput characterization of IRESes (Figure 1B) (21, 39). Our constructs consist of the splice acceptor upstream of a barcode, IRES, and firefly luciferase followed by the splice donor.

Our library, used throughout this work, consisted of 96 IRESes from a range of hosts and IRES structural types (Figure 1C) (39). Approximately half of the IRESes come from viruses with human as the primary host, while the rest span vertebrate hosts including amphibians, ungulates, and non-human primates. We confirmed by RT-qPCR that the library contained the circular topology (Figure S1).

Importantly, our library consisted of full length IRESes, spanning 365 nt to 1431 nt. This stands in contrast to previous studies that screened ~200 nt IRES fragments or with less IRESes (40–42). Representation of IRESes in both the template plasmid library and the circRNA library exhibited strong correlation using both random fragmentation and PCR amplicon sequencing methods (Figure S2).

## Type V IRES activity doubles that of the EMCV standard

We used polysome profiling to characterize the activity of our entire library in a single experiment (43). Using a sucrose gradient, we collected fractions corresponding to RNAs with one, two, three, etc. ribosomes (Figure 1D). Our optimized protocol enabled us to consistently recover highly resolved polysome fractions up to heptasomes across a variety of cell types (Figure 1E, S3). We then calculated the average number of ribosomes per RNA, a proxy for translation, using the frequency of barcode representation in each fraction (Figure 1F, G). Importantly, the barcode abundances in corresponding fractions are highly reproducible across biological and technical replicates (Figure S5).

**Figure 2:**
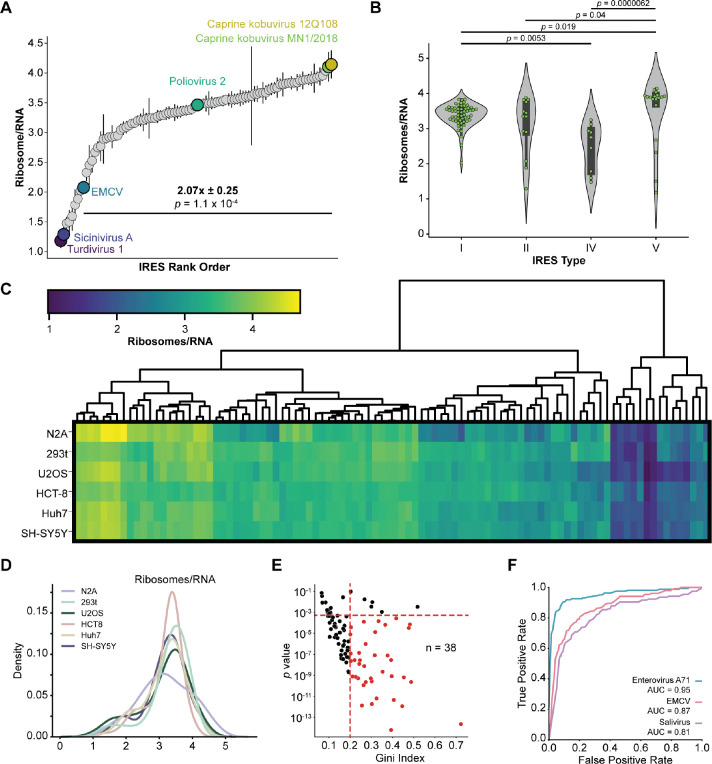
Type V IRESes double EMCV activity and most IRESes exhibit tissue tropism. (**A**) IRES activity (ribosomes/RNA) ranked across 96 IRESes in HEK293T cells. Select IRESes are labeled. EMCV activity relative to the top-performing Caprine kobuvirus 12Q108 is indicated. *p*-values from Student’s t-test, n = 4. (**B**) Ribosomes/RNA grouped by IRES structural type. p-values from Kruskal-Wallis H test with Dunn posthoc analysis and Bonferroni correction (n = 4). (**C**) Heatmap of ribosomes/RNA for all 96 IRESes across six cell types, with hierarchical clustering. Color scale indicates ribosomes/RNA. (n = 4/cell type). (**D**) Density distributions of IRES activity by cell type. (**E**) Tropism significance ( p-value) versus effect size (Gini Index) for each IRES. Dashed line indicates significance threshold after Bonferroni correction. 38 IRESes with moderate to strong tropism. *p*-values from ANOVA. (**F**) ROC curves for DMS-MaPseq constrained models of Enterovirus A71, EMCV, and Salivirus.

We initially performed polysome profiling in HEK293T cells, where IRES activity spanned approximately half an order of magnitude (Figure 2A). Notably, the EMCV IRES, typically used in synthetic constructs and gene therapy designs, exhibited comparatively poor performance (87^th^/96). Surprisingly, the top ten expressing IRESes in HEK293Ts were all Type V, from viruses with either ungulate or canine natural hosts. Type V is the most recently classified IRES, first identified in 2011, and remains the least studied of the five types, with only a handful of individual members functionally characterized to date (17).

To confirm that the polysome profiling data corresponded to an alternative method of translational readout we turned to a classic dual luciferase reporter assay (Figure 1A, S4). We tested the top two and bottom two performing IRESes, the median performing IRES (Poliovirus 2), and the EMCV IRES. All IRESes corresponded to the rank order from polysome profiling (Figure S4). Beyond rank order the two assays recapitulated relative expression (*e.g.* Caprine kobovirus 12Q108 had 2.07 ± 0.25 and 2.26 ± 0.25 fold activity compared to EMCV in polysome profiling and dual luciferase respectively).

## Most IRESes exhibit tissue-specific activity

Next, we profiled IRES activity in a total of six cell lines representing a range of tissues, namely HEK293T (renal), HCT-8 (colorectal), Huh7 (hepatic), N2A (neural murine), SH-SY5Y (neural), and U2OS (osteal) (Figure 2C, S3). To our knowledge, this atlas is the largest to date detailing such activity for full-length IRESes. The cell lines in these experiments represent a wide range of tissues, most of which are relevant in picornaviral infections. Crucially, this atlas is comprised of identical experimental, collection, and analysis procedures across the cell types ensuring the data is directly comparable and representative of cell type specific effects.

On a broad scale, the rank order of IRES activity was preserved between cell lines. For example, Type V IRESes performed equal to or better than the other IRES types in all cell lines we tested (Figure 2B, S6). This spoke to the primary regulatory mechanisms being common to all the cell types. These data are not surprising given IRESes are classified into a limited number of large scale structural types that necessarily serve as central scaffolds across many hosts and tissues (8). Nonetheless, IRESes that deviated from such ordering were identifiable in every cell comparison. This is in line with the fact that the different cell types had unique distributions of IRES activity profiles (Figure 2D).

We found that 77/96 IRESes displayed significant tropism (*i.e.* cell type specific activity) (Figure 2E). Using Gini Index (a measure of statistical dispersion) as a measure of effect size, we found 36/96 (~40%) exhibited moderate to strong significant tropism (Figure 2E). At the extreme end, Rhimavirus A had a 1.9 ± 0.1 fold expression (N2A/U2OS) and Seal Picornavirus type 1 had 1.6 ± 0.2 fold expression (293T/U2OS). Whereas, Caprine kobovirus 12Q108 has barely any tropism at 1.2 ± 0.2 fold expression (293T/HCT8).

## High-throughput modeling of IRES secondary structure

We then used DMS-MaPseq to generate experimentally informed *in vitro* and *in cellulo* structure models for our whole library (Figure S7) (44–46). We found that IRES structure mostly exhibited minimal conformational differences between circular and linear topologies (Figure S7C). Our Enterovirus A71, EMCV, and Salivirus models showed high agreement with published structures (Figure 2F) (47). Notably, *in vitro* and *in cellulo* data were correlated, indicating that IRES structure is largely encoded by sequence and thermodynamics rather than shaped by the cellular environment. This parallels observations in *E. coli* ribosomal RNA, where deproteinized rRNA folds to near-native structure *in vitro* (48). We provide DMS-MaPseq constrained structure models for all 96 IRESes at www.rnandria.org (27).

## IRES structure emerges from a language model trained on sequence alone

The complexity of IRES regulation evident in our functional atlas demanded structural explanations, but experimentally resolved structures exist for only a handful of IRESes.

**Figure 3:**
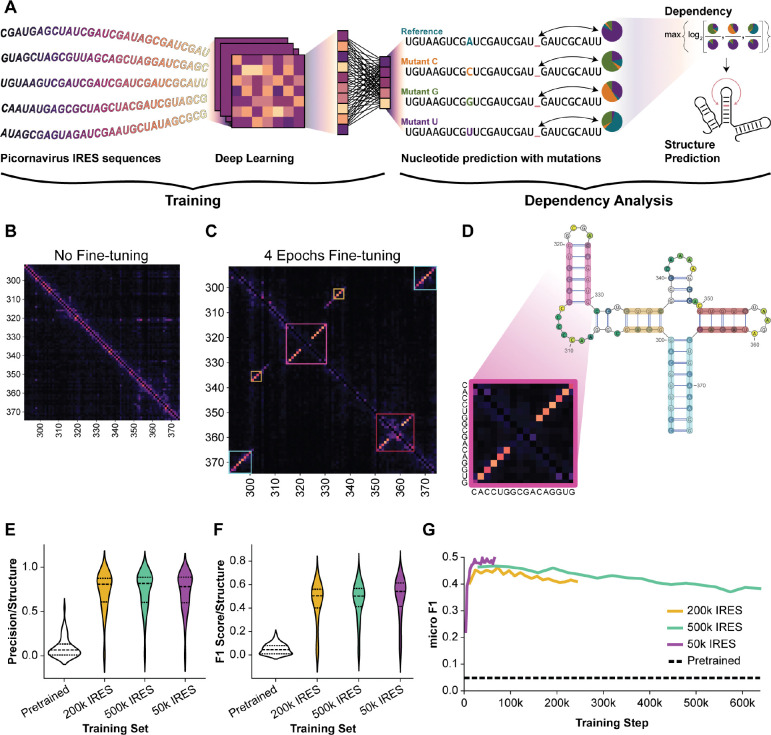
Albatross predicts IRES secondary structure from sequence alone with high precision. (**A**) Schematic of the dependency mapping approach. IRES sequences are used to fine-tune an RNA language model. For each position, the effect of every possible point mutation on nucleotide predictions is measured, generating a dependency map from which structure is inferred. (**B**) Dependency map of the EMCV IRES using the pre-trained model (no fine-tuning), showing no discernible structural signal. (**C**) Dependency map of the EMCV IRES after four epochs of fine-tuning. Antidiagonal features (dashed boxes) correspond to base-paired stems. (**D**) Albatross-predicted structure of the EMCV IRES overlaid on the experimentally validated secondary structure. Colors of the stems correspond to colored squares in C. Inset shows the dependency map for a stem region with corresponding sequence. (**E**) Precision per structure across training sets. (**F**) F1 score per structure across training sets. (**G**) MicroF1 over training steps for the 200k, 500k, and 50k training sets. Dashed line, pre-trained baseline.

Recently, da Silva et. al demonstrated that dependency mapping is able to predict secondary structure of common RNAs (*e.g.* tRNAs) using an LLM trained on sequences alone (34). LLMs capture relations between tokens (base identity in the case of RNA) in their higher-dimensional encoding. Dependency mapping uses a trained LLM to measure how the prediction of a given nucleotide depends on a single point mutation elsewhere in the sequence, revealing a variety of structural elements. This approach is, in part, motivated by covariation analysis. If a mutation in one base leads to a changed prediction in another base, this indicates base pairing interactions (Figure 3A).

Several pre-trained RNA Language Models exist (33, 49, 50). Unfortunately, these models were not trained on IRESes, and accordingly, performed poorly in our use case. For example, dependency mapping of the EMCV IRES returned noise (Figure 3B). Nonetheless, they capture some general patterns of RNA sequences so we decided to leverage this basis and fine-tune an RNA Language Model on IRES sequences.

We started from RiNALMo, a 650M-parameter base model pre-trained on 36M non-coding RNA sequences (33). Rather than training from scratch, we fine-tuned this model on IRES sequences, a transfer learning strategy widely employed in natural language processing and computer vision to adapt pre-trained models to new domains. We collected ~10,000 picornaviral 5′ UTRs from the NCBI Virus Database (51). For each, we performed a BLAST alignment against the nt database and collected the top 500 results (52). After deduplication, this yielded ~200,000 IRESes for fine-tuning RiNALMo. Importantly, training was done exclusively on RNA sequences with no explicit information on structure or base pairing rules. We dubbed the fine-tuned model Albatross.

Dependency mapping of the EMCV IRES using Albatross revealed antidiagonal dependencies corresponding to stems in the experimentally validated secondary structure (Figure 3C, D, S7) (47, 53).

## Smaller, high diversity training sets improve Albatross performance

To validate our dependency maps family-wide, we used our 96 DMS-MaPseq constrained structure models as “ground truth” (Figure 3D, S7). While these models are imperfect representations of reality, no other large-scale dataset of full-length IRES structures exists and the current state-of-the-art for mapping full-length IRESes remains chemical probing (42, 47, 54). Importantly, all of our models had AUC-ROC values between 0.83–0.98, indicating high agreement between the predicted structures and the underlying DMS reactivity data (Figure S7) (55, 56).

From the dependency maps, we generated explicit structures by applying a binary filter to assign base pairing (Methods). Our filtering did not specify base pairing rules, allowing for non-canonical base pairs. Across the 96 IRESes, Albatross achieved consistently high precision, with a median of ~0.78 and individual structures reaching as high as 0.98 (Figure 3E, S8, Table S1) (18,47,53,57). This means that when Albatross predicts a base pair, it is nearly always correct. High precision coupled with lower recall (microF1 ~0.45) is consistent with a model that preferentially captures structures maintained by selection while potentially missing thermodynamically stable but functionally neutral features (Figure 3F). This property makes the dependency maps particularly valuable for focusing on biologically relevant structural candidates. Notably, when we examined an apparent false positive where the model predicted stems absent from the DMS-constrained structure, manual inspection revealed that the model prediction was in fact correct (Figure S9). This indicates that the model’s true precision is likely higher than reported, and that we are approaching the ceiling of agreement achievable when benchmarking against imperfect reference structures.

We next sought to optimize training. We postulated that a greater training set could improve performance so we developed a larger training set, prioritizing the expansion of small clusters for a total of ~500,000 sequences. We compared the microF1 over 10 epochs of training (Figure 3G). While the 500k training set performed better than 200k, both rapidly reached a peak microF1 and worsened with longer training. The surprising degradation of performance indicated some form of over-training was occurring. We speculated that with more learning steps Albatross had worse scores through two mechanisms: 1) Albatross over-optimized for the highly represented sequences leading to worse performance on lowly represented sequences; 2) Albatross starts to forget its pretraining as we overtrain on the finetuning sequences (58).

We considered if a smaller but more diverse training set could recapitulate the “ground truth” structures better. We truncated our 500k training set down to ~50,000 sequences, prioritizing balanced inclusion from each >70% similarity cluster (Methods). This smaller library attained a substantially better microF1 than the two larger libraries (Figure 3F). In addition to improved performance, the smaller data set required less training, taking only twelve hours on one GPU. We evaluated F1 for each individual IRES using the best microF1 checkpoint for each training set. F1 scores trended upwards from the 200k to 500k to 50k training sets (Figure 3F). The best structure, for WUHARV Enterovirus 2, a Type I IRES, had an F1 of 0.74. Precision remained consistently high across all training sets, so we used the 50k model for the remainder of this work (Figure 3E).

**Figure 4:**
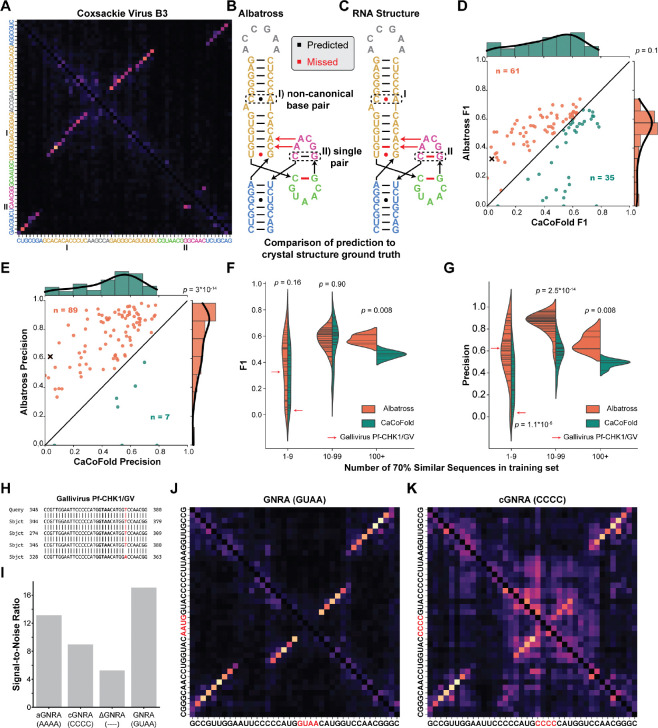
Albatross outperforms covariation analysis and uses motif recognition to predict structure. (**A**) Albatross dependency map of the Coxsackievirus B3 eIF4G-binding Domain V. (**B**) Albatross-predicted base pairs compared to the crystal structure, with predicted interactions in black and missed interactions in red. Albatross correctly identifies (I) a non-canonical A·C pair and (II) an isolated single G·C stabilizing interaction. (**C**) RNAstructure Fold thermodynamic prediction for the same region. (**D,E**) F1 and precision for Albatross versus CaCoFold across 96 IRESes *p*-values from a Mann-Whitney U test (n = 96). (**F,G**) F1 and precision binned by number of similar sequences in training set. *p*-values from a Mann-Whitney U test (n = 47, 44, 5, respectively). (**H**) BLAST alignment of the Gallivirus Pf-CHK1/GV GNRA region to the training data, showing only four related sequences. (**I**) Signal-to-noise ratio for GNRA stem in dependency maps of Gallivirus IRES with GNRA mutations. (**J,K**) Dependency maps of the Gallivirus IRES with GNRA mutations: wild-type (GUAA) and CCCC.

As an independent validation, we compared the Albatross-derived dependency map of the Coxsackievirus B3 IRES against the recently solved crystal structure of the eIF4G-binding domain V (59). Albatross correctly predicted 19 of 21 base pair interactions, outperforming the thermodynamics-based RNAstructure algorithm, which successfully identified only 16 interactions (Figure 4A–C). Notably, Albatross independently predicted both a non-canonical A • C pair and an isolated single G • C base pair stabilizing interaction first revealed by the crystal structure(Figure 4B, C). These findings demonstrate the potential for dependency maps to identify base interactions missed by conventional RNA secondary structure prediction approaches.

## Albatross dependency mapping outperforms covariation analysis

We wanted to compare Albatross with state-of-the-art covariation analysis and to that end we utilized CaCoFold to predict structures for the same 96 IRESes (32). We provided CaCoFold with the exact sequences used to train our 50k model as a reference and generated structure models for the 96 IRES in our library. We compared the F1, precision, and recall for each IRES (Figure 4D, E, S11A, Table S2). The difference in F1 score did not reach significance but trended in favor for Albatross. Importantly, in terms of precision, Albatross outperformed CaCoFold in 89/96 sequences (Figure 4E). We further allowed CaCoFold access to the full 500k sequences while still comparing to our 50k model and the results were largely similar (Figure S11B–D, Table S2).

When we binned IRESes by the number of sequences sharing at least 70% similarity, Albatross maintained high precision even for IRESes with few close relatives, whereas CaCoFold performance declined sharply (Figure 4F, G). One of the most striking cases was a highly divergent Gallivirus IRES whose GNRA elbow structure aligned to only four sequences in the training data, with a single mutation among them (Figure 4H). With so few related sequences, covariation analysis is effectively impossible, and accordingly CaCoFold failed to predict this structure. Yet Albatross predicted it correctly.

## Albatross predicts structure through motif recognition

If Albatross cannot rely on covariation for the Gallivirus IRES, it must be using a different strategy. We hypothesized that the model had learned to associate specific sequence motifs with structural outcomes, namely that GNRA tetraloops are consistently followed by elbow structures. To test this, we performed dependency mapping on the Gallivirus IRES with mutations in the GNRA motif: substitution to all As, all Cs, or complete deletion (Figure 4I–K, S10). The wild-type sequence produced clear dependencies corresponding to the elbow. Even a conservative GU to AA substitution introduced substantial noise in the GNRA stem, and substitution to CCCC further increased noise that propagated into the second stem of the elbow (Figure 4I, S10). Our filtering protocol could still reconstruct the correct structure despite this noise, but Albatross is clearly using the identity of the GNRA motif to predict the neighboring stem. Deletion of the GNRA motif eliminated the stem entirely, confirming that both motif identity and spacing drive the prediction (Figure S10B, C). These results demonstrate that Albatross has learned to associate linear sequence motifs with structural context, a capability that extends beyond what covariation analysis can access.

## Dependency mapping at scale identifies a Type II structural subclass

We ran ~75,000 unique IRES sequences through Albatross, generating a dependency map for each and manually labeling them by IRES type. These maps and predicted structures are freely available at www.albatrossrna.org. We then sought to detect structural variation within types using a spectral analysis pipeline (Methods) that assigned an “anomaly score” based on deviation from a canonical structural fingerprint approximating the median IRES for each type.

**Figure 5:**
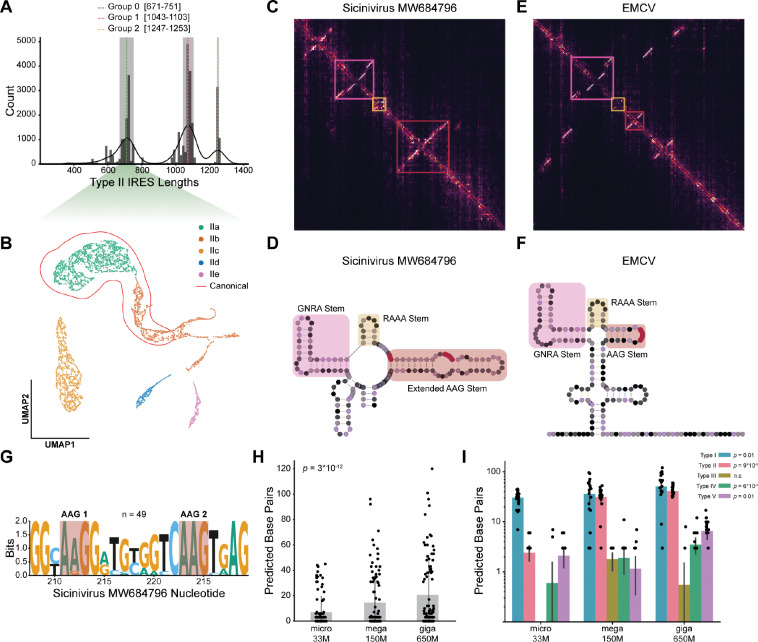
Albatross reveals structural variation within IRES types and generalizes across them. (**A**) Length distribution of Type II IRESes grouped into three bins. (**B**) UMAP of spectral analysis scores for 671–751 nt Type II IRESes, showing five clusters (IIa–IIe). “Canonical fingerprint” indicated. (**C,E**) Dependency maps for Sicinivirus MW684796 and EMCV, with structural features boxed. (**D,F**) Predicted structures for Sicinivirus and EMCV Domain I, highlighting GNRA stem, RAAA stem, and the extended AAG stem in the Sicinivirus variant. (**G**) Sequence logo of the extended AAG region across related sequences (n = 49). (**H**) Predicted base pairs scale with model size (33M to 650M parameters) on Type I IRESes. *p*-value calculated with Page’s trend test (n = 100/model). (**I**) Cross-type generalization: predicted base pairs by IRES type across model sizes. Models trained only on Type I sequences. *p*-values from Page’s trend test (n = 20/type/model).

We focused on Type II IRESes, which span multiple genera including cardioviruses and aphthoviruses, encompass EMCV, the most widely used IRES in bioengineering, and has recently been the subject of detailed structural and mechanistic studies of ribosome recruitment (53, 60, 61). We filtered the Type II dependency maps into three groups based on sequence length (Figure 5A). For the 671–751 nt sequences, spectral analysis revealed five clusters, which we designated IIa-IIe (Figure 5B). Cluster IIa sequences closely resembled the canonical fingerprint, while cluster IIb was the most heterogeneous (Figure 5B, S12).

As a test case, we investigated the most deviant IRES in cluster IIb, a Chicken Sicinivirus *sp.* with a high anomaly score. This IRES retained hallmark Type II features including the GNRA-containing Domain I and the eIF4G-binding Domains J-K, with Domain J exhibiting high sequence and structure identity to EMCV (Figure 5C, D). However, the Sicinivirus harbored an extended AAG stem in Domain I not present in canonical Type II IRESes (Figure 5C–F). This stem contains two conserved AAG motifs, with spacing from the RAAA stem to the first AAG matching that of EMCV, potentially enabling a similar contact with uS19 as recently reported (61). Both AAG motifs are conserved among related sequences, and several of these are predicted to fold with a similarly extended stem (Figure 5G). This structural variant, identified entirely through Albatross and spectral analysis, represents a previously unreported Type II subclass.

## Increasing model size leads to better prediction performance and generalization capabilities

To understand what drives improved dependency mapping performance, we leveraged the three available sizes of RiNALMo: mini (33M parameters), mega (150M), and giga (650M) (33). We fine-tuned each exclusively on Type I IRES sequences (~38,000) and evaluated them on 20 IRESes of each type, using the number of predicted base pairs as a proxy for performance, justified by the high precision of our approach.

A 20-fold increase in model size tripled the number of predicted base pairs, mirroring the scaling laws observed for language models in natural language processing (Figure 5H) (62). More striking was the effect on IRES types not seen during training. Although these models were fine-tuned only on Type I sequences, performance improved across all types with increased model size (Figure 5I). Type II predictions went from ~2 to ~41 base pairs, while Types IV and V rose from ~1 to ~4 and ~2 to ~7, respectively.

This cross-type generalization is particularly informative. The IRES types are structurally divergent, so the improved performance on Types II, IV, and V cannot be attributed to covariance with the Type I training data. Together with the Gallivirus mutational data (Figure 4F, H), these results demonstrate that Albatross develops generalized structural rules that scale with model capacity (Figure 5I). They also support recent crystallographic evidence for a shared structural logic among IRES types that Albatross is broadly capturing (59).

## Discussion

This work aimed to study picornaviral IRESes as a comprehensive family rather than as individual RNAs. In doing so, we generated large-scale functional (96 IRESes across 6 cell types) and structural (~75,000 dependency maps) atlases. These data are available in Supplementary Data S1 and at www.albatrossrna.org, respectively.

A recent study examining IRES context effects corroborates our activity rankings in overlapping cell types (42). Across our atlas, Type V IRESes consistently showed strong activity in all cell types tested, and most IRESes exhibited significant tissue tropism.

Using dependency mapping, we generated ~75,000 IRES structural maps. Albatross was trained on sequence alone, with no knowledge of structure or thermodynamics, yet predicted base pairs with high precision, outperforming state-of-the-art covariation analysis. This advantage likely stems from a fundamental limitation of covariation: it depends on accurate multiple sequence alignments, and picornaviral IRESes are notoriously divergent in primary sequence, making reliable alignment difficult. Dependency mapping bypasses this entirely, operating on individual sequences without alignment, making it inherently more robust for highly divergent RNA families.

Crucially, through multiple experiments, we demonstrate that the Albatross dependency mapping approach learns generalized patterns that it then can apply to divergent but related contexts (Figure 4F, G, 5I).

In analyzing the bulk data, we discover well-resolved structural groups among the Type II IRESes requiring further investigation beyond the scope of this work. Among these, we found a subclass of IRESes with an extended AAG stem. While we do not investigate the specific function of this variant Type II IRES, the identification of a candidate functional variation highlights the utility of dependency mapping for large scale RNA family analysis.

Overall, we provide a family-scale analysis of IRESes through multiple high-throughput avenues. Our functional data can be applied to biotechnology and infectious disease research investigating picornaviral tropism. Meanwhile, we establish RNA Language Model fine-tuning with dependency mapping as a viable approach for high-throughput structural analysis of diverse RNA families. This work demonstrates that sequence information is sufficient to generate high quality RNA structure prediction, and that increasing model size provides a path toward generalization.

## Supplementary Material

Supplement 1

Supplement 2

Materials and Methods

Figures S1 to S12

Tables S1 to S2

References *(64–66)*

Data S1

## Data Availability

Code, model weights, and training data are available at https://github.com/rouskinlab/. DMS-MaPseq data is available at www.rnandria.org. Albatross dependency maps and visualization are available at www.albatrossrna.org. Physical materials are available by request. .
