# Supplementary material for: An RNA Language Model trained on sequence alone reveals the structural logic of Internal Ribosome Entry Sites
Source: bioRxiv. 2026 May 20:2026.05.19.726202. Preprint. [Version 1] doi: 10.64898/2026.05.19.726202 (PMC13228543; doi:10.64898/2026.05.19.726202)
Supplement: Supplement 2 [file NIHPP2026.05.19.726202v1-supplement-2.pdf]

bioRxiv preprint doi: https://doi.org/10.64898/2026.05.19.726202; this version posted May 20, 2026. The copyright holder for this preprint (which was not certified by peer review) is the author/funder, who has granted bioRxiv a license to display the preprint in perpetuity. It is made available under a CC-BY 4.0 International license.

Supplementary Materials for

An RNA Language Model trained on sequence alone reveals the structural logic of Internal Ribosome Entry Sites

Adam Sychla[1†], Pierre Bongrand[1†], Grant Yang[1,2], Jacob Rulison[1,2], R. Alexander Wesselhoeft[3], Namita Bisaria[4], Silvi Rouskin[*,1]

[*]Corresponding author. Email: silvi@hms.harvard.edu

[†]These authors contributed equally to this work.

This PDF file includes:

Materials and Methods

Figures S1 to S12

Tables S1 to S2

Captions for Data S1

Other Supplementary Materials for this manuscript:

Data S1

bioRxiv preprint doi: https://doi.org/10.64898/2026.05.19.726202; this version posted May 20, 2026. The copyright holder for this preprint (which was not certified by peer review) is the author/funder, who has granted bioRxiv a license to display the preprint in perpetuity. It is made available under a CC-BY 4.0 International license.

Materials and Methods

Plasmids

All PCR reactions were performed using CloneAmp HiFi PCR Premix (Takara Cat. #639298). Isothermal Assembly was performed using NEBuilder HiFi DNA Assembly Master Mix (NEB E2621L). Constructs were chemically transformed into NEB Stable Competent E. coli (NEB C3040I) or electroporated into NEB 10-beta Competent E. coli (NEB C3019I).

RT-qPCR

cDNA was synthesized using LunaScript RT SuperMix (NEB M3010L). RT-qPCR was performed using either either Luna Universal Probe qPCR Master Mix (NEB M3004L) for probe-based or Luna Universal qPCR Master Mix (NEB M3003) for SYBR-based RT-qPCR as noted throughout.

Next-generation sequencing

Next-generation sequencing library preparation was performed using either NEBNext UltraExpress RNA Library Prep Kit (NEB E3330L) or NEBNext UltraExpress DNA Library Prep Kit (NEB E3325L) as appropriate. Next-generation sequencing was performed on a Illumina NextSeq using NextSeq 1000/2000 P1 XLEAP-SBS Reagent Kit (300 Cycles) (Illumina 20100982).

circRNA Plasmid Library

IRES sequences were obtained from Twist Bioscience in 96-well plate format. Each IRES included overhangs to clone into the circRNA generation plasmid provided by the Mass General Hospital RNA Therapeutics Core. The donor circRNA plasmid (p10-Natural-RTC) was amplified using and primers oAS092 and oAS093. IRES and donor plasmid were assembled together using isothermal assembly and transformed into NEB 10-beta Competent E. coli (NEB C3019I).

The distribution of IRESes within the plasmid library was confirmed by Next-Generation Sequencing a PCR amplicon of the barcode region associated with each IRES (Primers oAS052, oAS077) (Figure S2A). These data recapitulated the results of sequencing randomly fragmented library by NEBNext dsDNA Fragmentase (NEB M0348S).

bioRxiv preprint doi: https://doi.org/10.64898/2026.05.19.726202; this version posted May 20, 2026. The copyright holder for this preprint (which was not certified by peer review) is the author/funder, who has granted bioRxiv a license to display the preprint in perpetuity. It is made available under a CC-BY 4.0 International license.

circRNA library generation and analysis

circRNAs were generated by the Mass General Hospital RNA Therapeutics Core. Distribution within the circRNA library was measured by generating cDNA then sequencing a PCR amplicon of the barcode region. This was validated by sequencing a randomly fragmented library (Figure S2B). Circularization was confirmed by probe-based RT-qPCR using primers spanning the splice junction (Figure S1).

Cell culture

All cell lines were grown in DMEM (ThermoFisher Scientific #10564011) with 10% FBS (ThermoFisher Scientific #A5209402) supplementation at 37 ^{o}C with 5% CO_2

Polysome profiling

Polysome gradient buffer (PGB) consisted of 20mM Tris-HCl pH 7.5 (Rockland #MB-002), 100mM KCl (Invitrogen AM9640G), 10mM $MgCl_2$ (Thermo Scientific J62411.AD). 10% and 50% sucrose solutions in PGB were set up. 13.2 mL Open-Top Thinwall Ultra-Clear Ultracentrifuge Tubes (Beckman Coulter 344059) were loaded with the 10% sucrose solution up to the level indicated by the BioComp Marker Block (BioComp Instruments 105-914A-R). Using a syringe attached to cannula the 50% sucrose solution was loaded to the bottom of the tube until the total solution was level with the BioComp Gradient Station MagnaBase Tube Holder (BioComp Instruments 105-914A-R). The tubes were capped and the SW41 Rotor, Long Sucrose 10-50% L11 protocol was run on the BioComp Gradient Station (BioComp Instruments 153).

10 cm plates containing transfected cells were treated with 100 μg/mL cycloheximide (Sigma-Aldrich C4859-1ML) for 10 minutes at 37 ^{o}C. Lysis buffer was made with 1 mL PGB, 500 μg cycloheximide (Sigma-Aldrich C4859-1ML), 5 μL NP-40 (BioBasic NDB0385-100ml). 500 μL of lysis buffer was added to each 10 cm plate. Cells were scraped and transfered in to 1.7 mL tubes on ice for 10 minutes. The lysis was spun down at 4 ^{o}C at 11,200 rcf for 5 minutes and supernatant collected into clean tubes.

150 μL of the lysate was loaded on to each sucrose gradient. The gradient was spun in a SW 41 Ti Rotor (Beckman Coulter 331362) in an L100XP ultracentrifuge (Beckman Coulter 392052)

bioRxiv preprint doi: https://doi.org/10.64898/2026.05.19.726202; this version posted May 20, 2026. The copyright holder for this preprint (which was not certified by peer review) is the author/funder, who has granted bioRxiv a license to display the preprint in perpetuity. It is made available under a CC-BY 4.0 International license.

at 36,000 rpm (221,356 rcf) for 1 hour 20 minutes.

Sucrose gradients were fractionated using the BioComp Gradient Station (BioComp Instruments 153) collecting fractions that corresponded to polysome peaks.

RNA was extracted using 1 mL TRIzol Reagent (TheroFischer Scientific 15596026) then following manufacturer protocol. cDNA was synthesized as above and barcodes were amplified via PCR reactions using CloneAmp HiFi PCR Premix (Takara Cat. #639298) for 25 cycles. Amplicons were sequenced by next-generation sequencing as above. RNA from the lysis was similarly processed.

Barcodes counts were normalized to the total reads then normalized to the fractional representation in the lysis.

$$\frac{Ribosome}{RNA} = \sum NormalizedReads * PolysomeNumber$$

Luciferase assays

Cells in a 96 well plate were transfected with 10 ng plasmid DNA via PEI protocol (MedChem Express HY-K2014). Twenty four hours later, media was removed and 80 uL of 1x Passive Lysis Buffer (Promega Cat.# E1910) was added to the cells. Luciferase assays were performed using the Nano-Glo Dual-Luciferase Reporter Assay System (Promega Cat.# N1610) according to manufacturer protocol.

DMS-MaPseq

For *in vitro* DMS-MaPseq, RNA was suspended to 0.5 μg/μL in 10 L of H2O, incubated at 95°C for 1 min, immediately mixed with 87 μL of 37 ^{o}C preheated refolding buffer (0.4M Sodium cacodylate (Electron Microscopy Sciences #11655), 6 mM MgCl2 (Thermo Scientific J62411.AD)). The reaction was incubated at 37 ^{o}C for 30 min. 3% DMS (Sigma-Aldrich #D186309-100ML) was added to each reaction and incubated at 37°C for 5 min before being quenched with 0.6 volumes β-Mercapethanol (Thermo Scientific A15890.0B). RNA was isolated using Monarch Spin RNA Cleanup Kit (NEB T2030L). Next-generation sequencing library preparation was performed using either NEBNext UltraExpress RNA Library Prep Kit (NEB E3330L) but replacing the reverse transcriptase with Induro Reverse Transcriptase (NEB M0681L).

bioRxiv preprint doi: https://doi.org/10.64898/2026.05.19.726202; this version posted May 20, 2026. The copyright holder for this preprint (which was not certified by peer review) is the author/funder, who has granted bioRxiv a license to display the preprint in perpetuity. It is made available under a CC-BY 4.0 International license.

For *in cellulo* DMS-MaPseq, 5 μg RNA was transfected into HEK293T in 6-well plates. After 6 hours, cells were treated with 3% DMS (Sigma-Aldrich #D186309-100ML) for 5 minutes at 37 ^{o}C. The reaction was quenched with 10 mL 30% β-Mercapethanol (Thermo Scientific A15890.0B) in DPBS (ThermoFisher Scientific #14190144). RNA was isolated using TRIzol extraction and libraries were prepared as described above.

DMS-MaPseq sequencing data was processed using SEISMIC v24.3 (`https://github.com/rouskinlab/seismic-rna`) to map DMS reactivity, cluster alternative structures, and evaluate secondary structure models (*46*).

RNA Language Model

We used as a base model RiNALMo. The model comes in three model sizes: micro, mega and giga. For every experiment besides 5 we used giga, a 650 Million parameter RNA Language Model.

RiNALMo's architecture was not modified. It is composed of 33 transformer blocks, 1280 embedding dim, 20 attention heads. It uses Flash attention. The attention dropout and residual dropout are both set at 0.1. RiNALMo was originally trained on 36M non-coding RNAs, none of which are IRESes.

The tokenization was the same as in the original RiNALMo. Each nucleotide is a single token. After collecting the IRESes two transformations were applied: First, the sequence would be uppsercased. Second, uracils were converted to thymines. These transformations were applied to stay in line with RiNALMo's pretrainining. Therefore, the Albatross vocaulary is made of 4 vocabulary tokens: A, C, G, T; and four special tokens: [CLS], [EOS], [PAD], [MASK], and [UNK].

Training data & Preprocessing

Initially we collected the 5′ UTRs of every complete picornaviral genome in the NCBI Virus Database (*51*). We then iteratively used BLAST to find the top 500 aligning sequences until we had a total of about 200,000 unique sequences (*52*). To make the 500,000 sequence dataset, we used cd-hit to cluster sequences by 70% or greater sequence similarity (*63*). We then ran an iterative BLAST on the least represented sequences until we had a total of about 500,000 unique sequences. The 50,000 sequence dataset was generated by using cd-hit to cluster the 500,000 dataset by 70%

bioRxiv preprint doi: https://doi.org/10.64898/2026.05.19.726202; this version posted May 20, 2026. The copyright holder for this preprint (which was not certified by peer review) is the author/funder, who has granted bioRxiv a license to display the preprint in perpetuity. It is made available under a CC-BY 4.0 International license.

or greater sequence similarity (*63*). The representative sequence of each cluster was added to the dataset, then the most divergent sequence of each cluster was added one at a time until the set included about 50,000 sequences.

Sequences collected containing any IUPAC ambiguity code were filtered out. Sequences having an exact match to another sequence were also filtered out, in order to avoid overrepresention.

The three datasets used in figure 4 are respectively composed of 193,643, 509,944, and 52,129 unique sequences. Three datasets were used throughout this paper; they are available on `https://github.com/rouskinlab/`.

Model Training

We downloaded the official released weights from RiNALMo and performed a full fine-tuning on all 650M parameters. We did not use any LoRA, adapters, or layer freezing. The full fine-tuning was done with sequence input only. No structural labels, base pairing rules or thermodynamic priors were given to the model or trained on. In that regard, we can see this fine-tuning, as a continued pretraining on the sequences of interest.

The Objective is the same one as used in RiNALMo: a BERT-style masked language modeling at 15% mask rate. This mask would then have the following distribution: 80% [MASK] / 10% random nucleotide / 10% unchanged.

The optimizer used was AdamW with parameters: $\beta = (0.9, 0.98)$, $\varepsilon = 10^{-6}$, learning rate was 5×10^{-6} and weight decay was 0.01 (*64*).

A linear warmup was set over 10,000 steps from $0.01 \cdot \mathrm{lr}$ to lr. There is no decay phase, the learning rate is constant after warmup.

The training parameters were: Batch size = 8, Max sequence length = 2048, Gradient clipping 1.0, 16-mixed precision, Random seed 42, Up to 10 epochs.

Every experiment was run on a single L40 GPU. The 50k run completed in approximately twelve hours.

The weights released with this paper are from the 50k dataset, epoch 5, step 39,096. All dependency maps used in this paper and available on `www.albatrossrna.org` were produced from this checkpoint.

bioRxiv preprint doi: https://doi.org/10.64898/2026.05.19.726202; this version posted May 20, 2026. The copyright holder for this preprint (which was not certified by peer review) is the author/funder, who has granted bioRxiv a license to display the preprint in perpetuity. It is made available under a CC-BY 4.0 International license.

Dependency Map Generation

The Methodology used to get the dependency map is the same as described in da Silva et al. (*34*).

For a sequence of length N, we run $3N + 1$ forward passes (1 for the reference sequence and every single-point substitution to each of the 3 alternative bases). For each of these forward passes, we extract softmax probability over $\{A, C, G, U\}$ at every position j. For mutation at position i to base m and target base t, compute the log-odds shift

$$S(i, j, m, t) = \log_2 \tfrac{p_{\text{mut}}}{1-p_{\text{mut}}} - \log_2 \tfrac{p_{\text{ref}}}{1-p_{\text{ref}}}, \tag{S1}$$

where $p_{\text{mut}} = P(x_j = t \mid i \to m)$ and $p_{\text{ref}} = P(x_j = t \mid \text{wild type})$ are the model's softmax probabilities at position j for target base t, under the mutant and reference sequences respectively. The dependency map entry is $M[i, j] = \max_{m,t} |S(i, j, m, t)|$, with $M[i, i] = 0$.

Binary Structure Filtering

Binary filtering and structure prediction consisted of the following algorithm:

Algorithm 1: Binary structure filtering from a dependency map.

Input: dependency map $M \in \mathbb{R}^{N\times N}$; thresholds τ, γ, α.

Output: binary contact matrix $B \in \{0, 1\}^{N\times N}$.

1. Normalize: $M' \leftarrow \text{clip}(M/20, 0, 1)$.

2. Symmetrize: $M' \leftarrow \max(M', M'^{\top})$.

3. Threshold: $M'[i, j] \leftarrow 0$ where $M'[i, j] < \tau$.

4. Diagonal-band exclusion: $M'[i, j] \leftarrow 0$ where $|i - j| \leq \gamma$.

5. Anti-diagonal connectivity: along each anti-diagonal $\{(i, j) : i + j = s\}$, retain only entries belonging to a contiguous run of length $\geq \alpha$.

6. Maximum-weight matching: build an undirected graph with edge weights $M'[i, j]$ and solve $B \leftarrow \arg\max_{\mathcal{M}} \sum_{(i,j)\in\mathcal{M}} M'[i, j]$ via Edmonds' Blossom algorithm. This enforces one-to-one pairing and allows non-canonical pairs.

bioRxiv preprint doi: https://doi.org/10.64898/2026.05.19.726202; this version posted May 20, 2026. The copyright holder for this preprint (which was not certified by peer review) is the author/funder, who has granted bioRxiv a license to display the preprint in perpetuity. It is made available under a CC-BY 4.0 International license.

7. Pseudoknot removal: sort accepted pairs by span $|j - i|$ in decreasing order, then iterate; retain a pair only if it does not cross any already-retained pair.

As Blossom matching can return crossing pairs, we removed pseudoknots to yield strictly nested structures representable in dot-bracket notation.

The filtering applied on the dependency map only encodes geometric priors and no biological priors. It is a significant design choice as it makes our prediction entirely dependent on the LLM log odds shifts. Therefore, the model has no idea of base pairing rules or thermodynamic priors. The dependency maps are raw and not symmetrized.

Each of the parameter was designed to encode for a different prior: α forces stem to have a minimum length. This prevents the filter to select isolated base pairs that also can be noise from the dependency map. γ excludes the base pairs immediately next to the diagonal, as these pairs cannot form (Triangle inequality). τ is the most significant parameter as it is the threshold to filter out weak signal.

Filter Parameter Selection (5-fold cross-validation)

In order to select the best set of parameters, we ran a 5-fold cross validation with a grid search on the 96 DMS-MaPseq-constrained IRES structures.

The 5-fold cross validation ran under the following settings: The 96 reference sequences were split using `KFold` with $K = 5$, `shuffle=True`, and `random_state=42`. For each fold, true positives, false positives, and false negatives were aggregated across the held-out sequences, and a micro-F1 score was computed. We then reported the mean and standard deviation across folds. The grid search covered $\alpha \in \{3, \ldots, 8\}$, $\tau \in [0, 0.20]$, and $\gamma \in \{3, 4, 5\}$, with micro-F1 used as the metric to evaluate each parameter combination.

Selected parameters and performance (50k, epoch 5).

The selected parameters were $\alpha = 3$, $\tau = 0.11$, and $\gamma = 4$. With these values, the model achieved a micro-F1 of 0.519 ± 0.042, a precision of 0.715 ± 0.060, and a recall of 0.407 ± 0.033.

bioRxiv preprint doi: https://doi.org/10.64898/2026.05.19.726202; this version posted May 20, 2026. The copyright holder for this preprint (which was not certified by peer review) is the author/funder, who has granted bioRxiv a license to display the preprint in perpetuity. It is made available under a CC-BY 4.0 International license.

Genome-Scale Structure Maps

Across the full set of binary maps, we observed a mean of 71.5 base pairs per map, a median of 78, and a maximum of 171. After substring-containment deduplication of the 500k pool, 75,229 root sequences were retained and used for the family-wide structural analyses. These structures can be found on https://albatrossrna.org.

Spectral Analysis

Each dependency map analyzed was transformed, mapping each diagonal of the $n \times n$ map to a left aligned row of a $(2n-1) \times n$ matrix. The transformed map was analyzed to detect the number of stems (N_s) and longest stem (L_s) along each row (i.e. diagonal). The intensity weighted average signal position (C_s) and drift from row to row (C_d), the symmetry of the row (S), and the sum of the squares of the signal for each row (E_r) were also calculated. A similar sum of squares was done column-wise (E_c). Finally, a radially binned 2D power spectrum was calculated (P_s, accounts for periodicity of the data along both dimensions to enable alignment free comparison of signal distribution).

A template "canonical IRES fingerprint" was generated with the median values of each above feature. For each map and feature, a difference was computed from the fingerprint. The features were Z-normalized and a final anomaly score computed as the L2-norm of the feature eight-vector. The UMAP represents a 2D projection of eight-vector, clustered by HDBSCAN (*65, 66*).

bioRxiv preprint doi: https://doi.org/10.64898/2026.05.19.726202; this version posted May 20, 2026. The copyright holder for this preprint (which was not certified by peer review) is the author/funder, who has granted bioRxiv a license to display the preprint in perpetuity. It is made available under a CC-BY 4.0 International license.

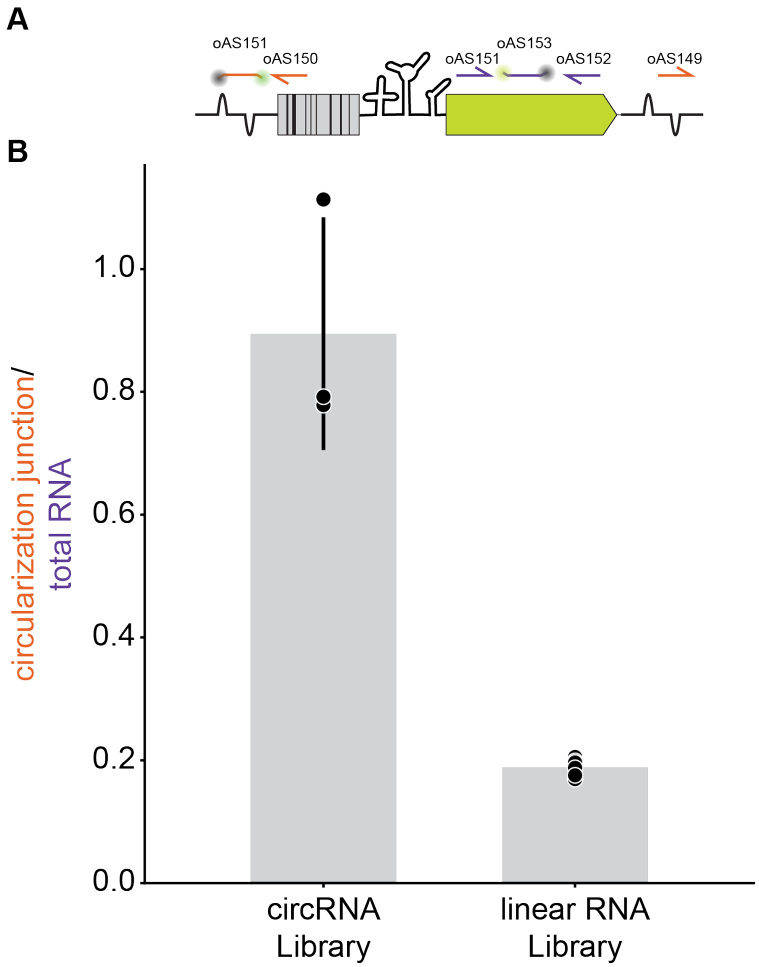

Figure S1: **RT-qPCR confirms circular topology of the circRNA library.** (A) Schematic of the RT-qPCR primer design. Divergent primers (oAS150–oAS153) span the circularization junction, with oAS149 targeting the linear backbone. (B) Ratio of circularization junction signal to total RNA for the circRNA library and the linear RNA library. The circRNA library shows high junction enrichment (~0.85), confirming efficient circularization, while the linear RNA library shows minimal junction signal (~0.17). Data points represent individual replicates; bars indicate the mean.

bioRxiv preprint doi: https://doi.org/10.64898/2026.05.19.726202; this version posted May 20, 2026. The copyright holder for this preprint (which was not certified by peer review) is the author/funder, who has granted bioRxiv a license to display the preprint in perpetuity. It is made available under a CC-BY 4.0 International license.

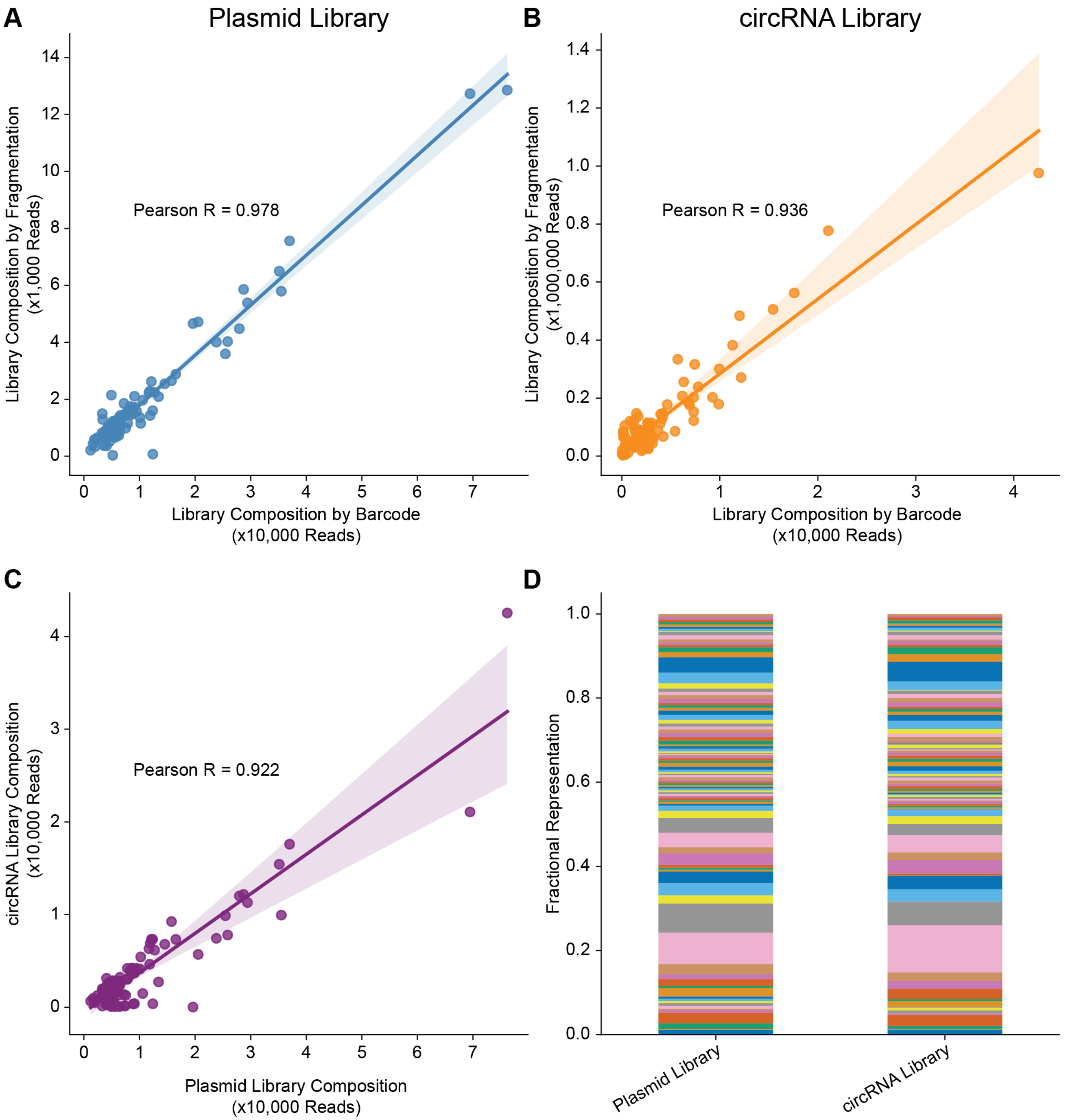

Figure S2: **IRES representation is consistent across library preparation and sequencing methods.** (A) Correlation between barcode sequencing and random fragmentation sequencing for the plasmid library (Pearson R = 0.978). (B) Same as (A), but for the circRNA library (Pearson R = 0.936). (C) Correlation between plasmid and circRNA library composition measured by barcode sequencing (Pearson R = 0.922). Each point represents one IRES. (D) Fractional representation of individual IRESes in the plasmid and circRNA libraries. Each colored segment corresponds to a single IRES. Shading is consistent between libraries. Lines indicate linear regression fits with 95% confidence intervals.

bioRxiv preprint doi: https://doi.org/10.64898/2026.05.19.726202; this version posted May 20, 2026. The copyright holder for this preprint (which was not certified by peer review) is the author/funder, who has granted bioRxiv a license to display the preprint in perpetuity. It is made available under a CC-BY 4.0 International license.

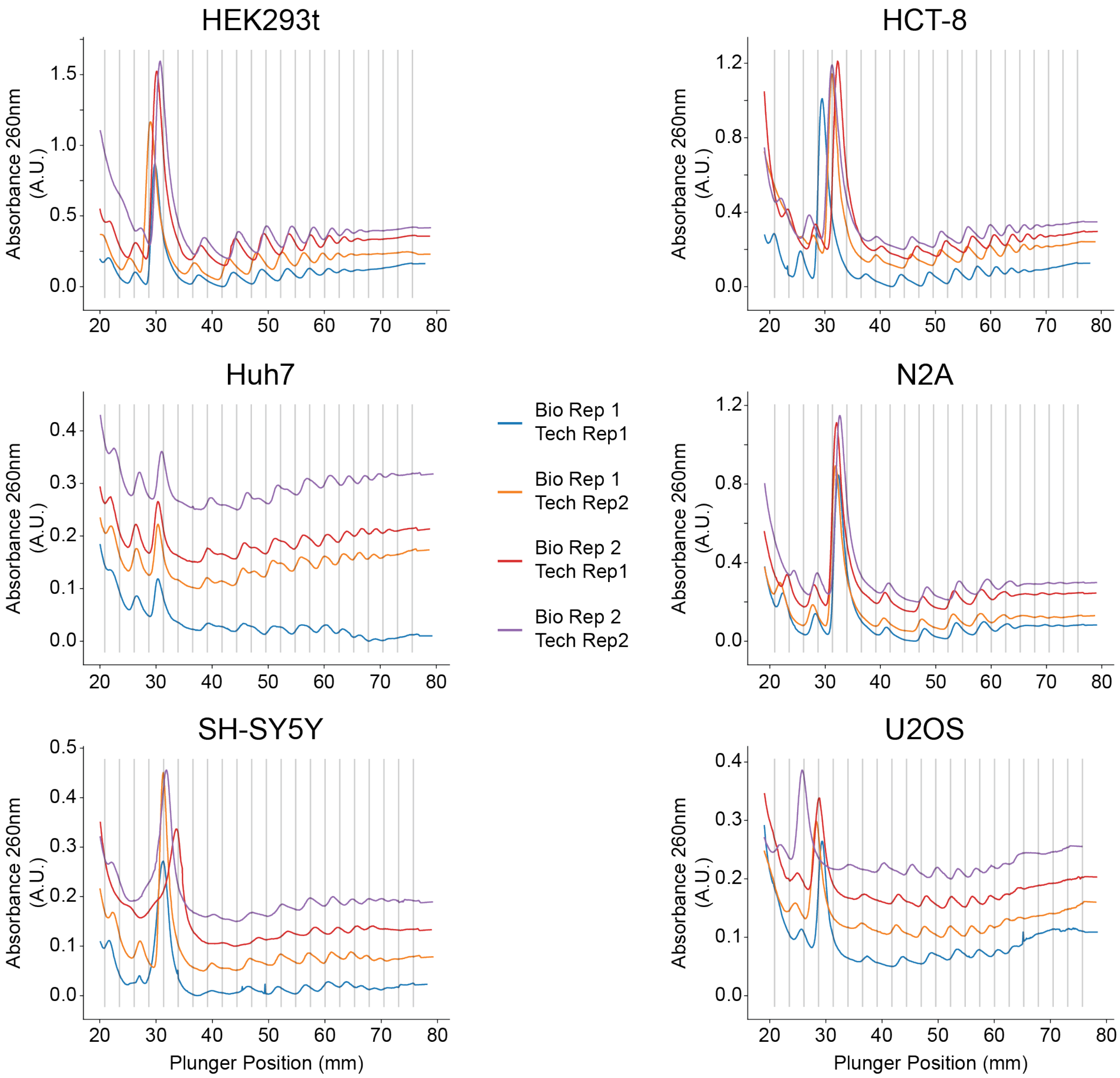

Figure S3: **Polysome fractionation absorbance at 260nm.** Trace of 260 nm absorbance during polysome fractionation. For each sample, absorbance was shifted equally at each point to set minimum absorbance to 0 A.U. Replicates were similarly shifted upwards for clarity. Vertical lines delineate fractions.

bioRxiv preprint doi: https://doi.org/10.64898/2026.05.19.726202; this version posted May 20, 2026. The copyright holder for this preprint (which was not certified by peer review) is the author/funder, who has granted bioRxiv a license to display the preprint in perpetuity. It is made available under a CC-BY 4.0 International license.

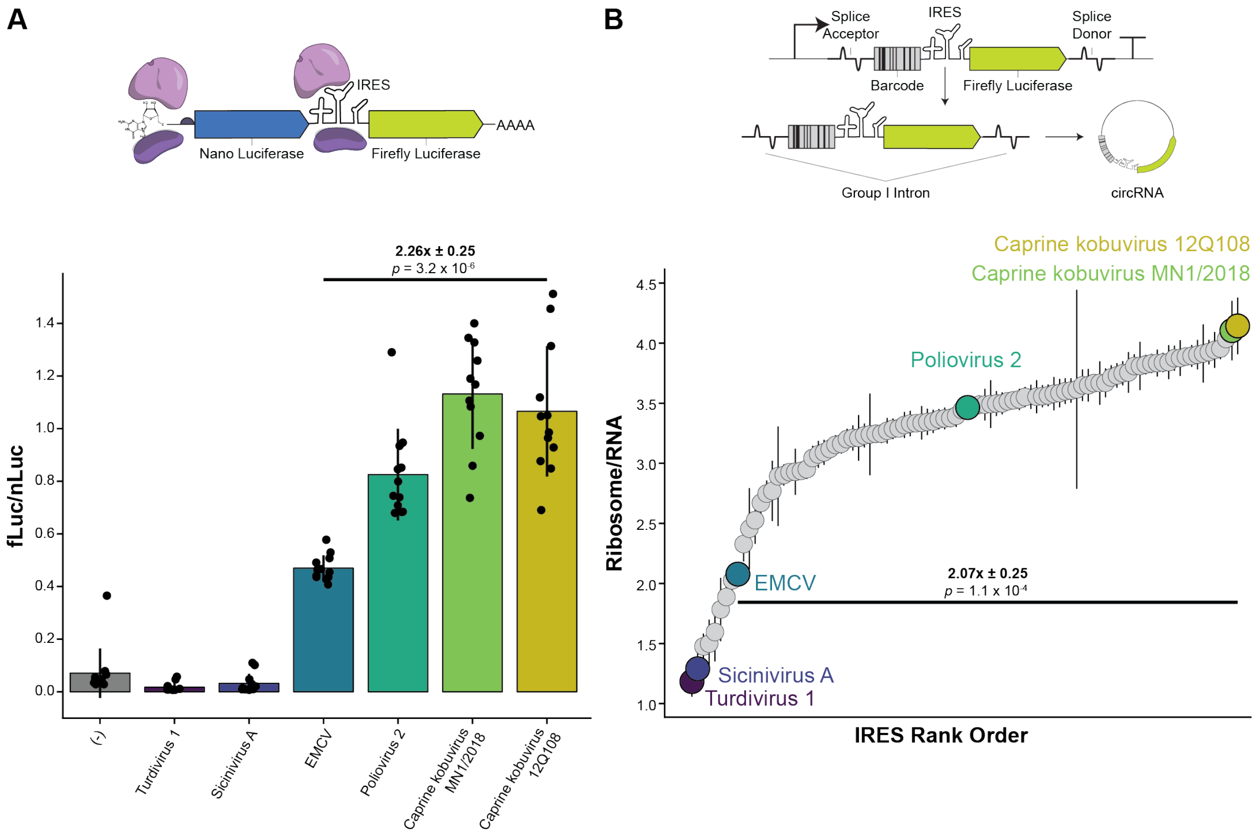

Figure S4: **Dual luciferase reporter assay validates polysome profiling rank order.** (A) Bicistronic dual luciferase reporter assay schematic (left) and firefly to nano luciferase ratios (fLuc/nLuc) for six selected IRESes: the top two (Caprine kobuvirus 12Q108, Caprine kobuvirus MN1/2018), bottom two (Turdivirus 1, Sicinivirus A), median (Poliovirus 2), and EMCV. Caprine kobuvirus 12Q108 exhibits 2.26 ± 0.25 fold activity relative to EMCV ($p = 3.2 \times 10^{-5}$). Data points represent individual replicates; bars indicate the mean. (B) CircRNA construct schematic (top) and ribosomes/RNA from polysome profiling for all 96 IRESes ranked by activity, with the same six IRESes highlighted. Caprine kobuvirus 12Q108 exhibits 2.07 ± 0.25 fold activity relative to EMCV ($p = 1.1 \times 10^{-3}$). Rank order and relative expression are concordant between the two assays. All data from HEK293T cells.

bioRxiv preprint doi: https://doi.org/10.64898/2026.05.19.726202; this version posted May 20, 2026. The copyright holder for this preprint (which was not certified by peer review) is the author/funder, who has granted bioRxiv a license to display the preprint in perpetuity. It is made available under a CC-BY 4.0 International license.

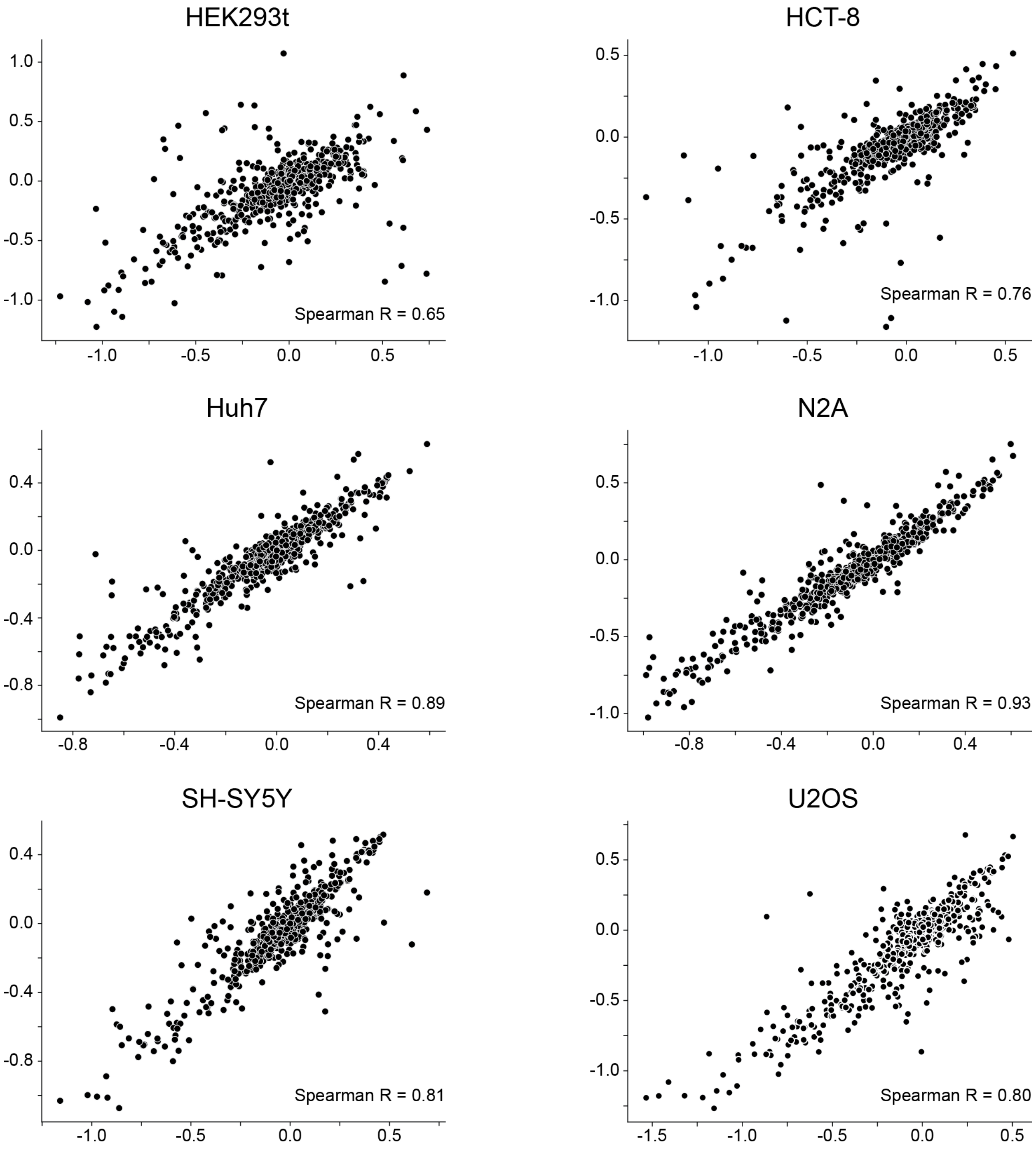

Figure S5: **Polysome profiling barcode abundances are reproducible across replicates.** Scatter plots of barcode abundances between biological replicates for each cell type. Spearman correlation coefficients are indicated: HEK293T (R = 0.65), HCT-8 (R = 0.76), Huh7 (R = 0.89), N2A (R = 0.93), SH-SY5Y (R = 0.81), and U2OS (R = 0.80). Each point represents one IRES barcode in a given polysome fraction.

bioRxiv preprint doi: https://doi.org/10.64898/2026.05.19.726202; this version posted May 20, 2026. The copyright holder for this preprint (which was not certified by peer review) is the author/funder, who has granted bioRxiv a license to display the preprint in perpetuity. It is made available under a CC-BY 4.0 International license.

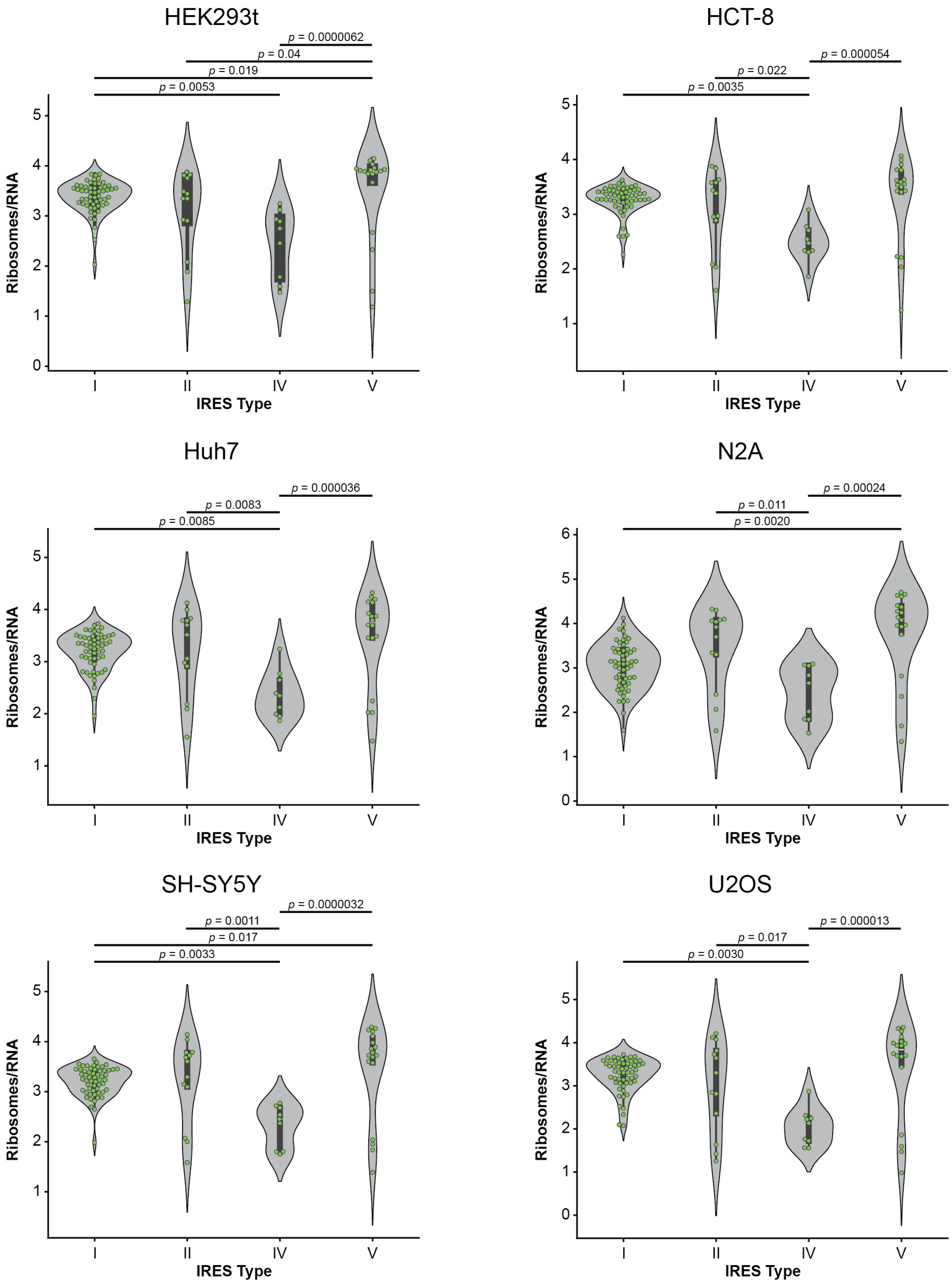

Figure S6: **Type V IRESes exhibit high activity across all cell types.** Same as Figure 2B, but shown individually for all six cell lines: HEK293T, HCT-8, Huh7, N2A, SH-SY5Y, and U2OS. Violin plots display ribosomes/RNA grouped by IRES structural type (I, II, IV, V). *p*-values from Kruskal-Wallis H test with Dunn posthoc analysis are indicated.

bioRxiv preprint doi: https://doi.org/10.64898/2026.05.19.726202; this version posted May 20, 2026. The copyright holder for this preprint (which was not certified by peer review) is the author/funder, who has granted bioRxiv a license to display the preprint in perpetuity. It is made available under a CC-BY 4.0 International license.

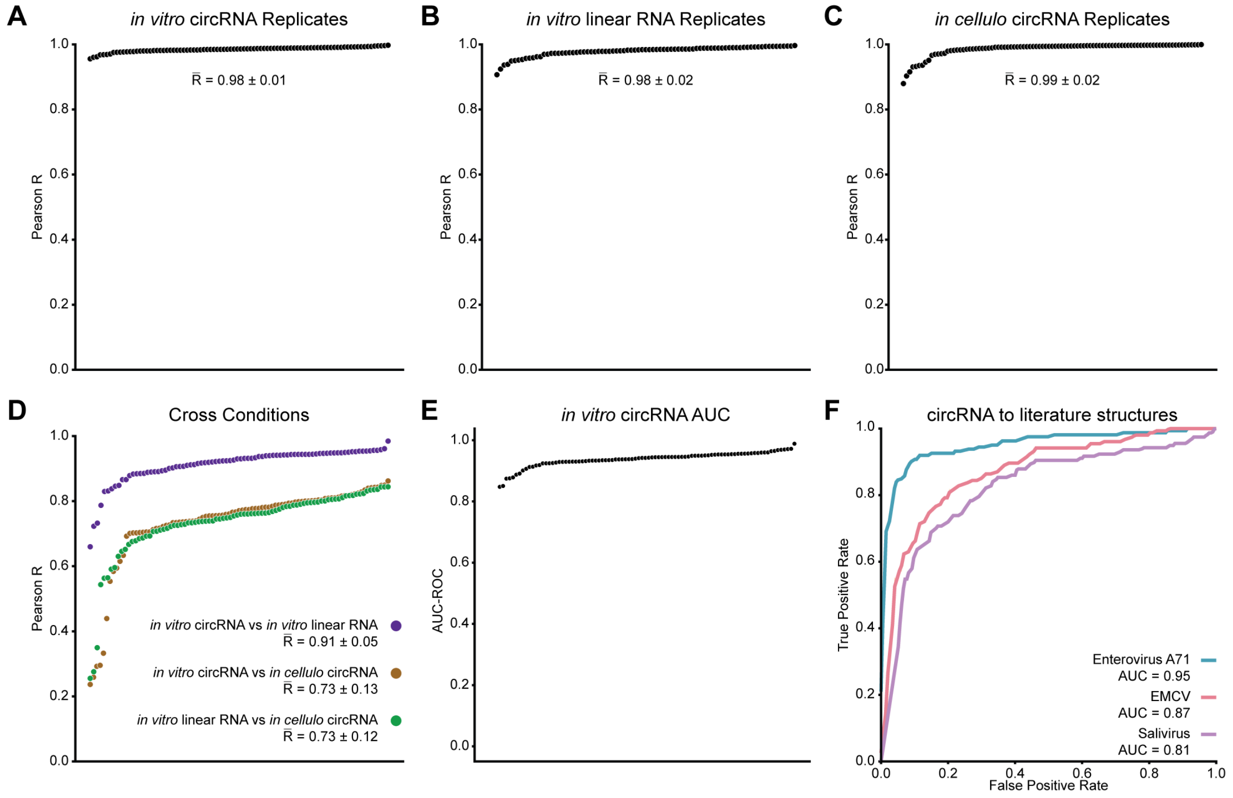

Figure S7: **DMS-MaPseq structure probing of the full IRES library.** (A–C) Replicate correlations of DMS reactivity for *in vitro* circRNA (A), *in vitro* linear RNA (B), and *in cellulo* circRNA (C) conditions. Pearson R values are indicated. (D) Cross-condition correlations comparing DMS reactivities between *in vitro* circRNA, *in vitro* linear RNA, and *in cellulo* circRNA, showing minimal conformational differences between topologies and conditions. (E) AUC-ROC distributions for DMS-MaPseq constrained structure models across conditions, confirming high agreement between predicted structures and underlying reactivity data (AUC range 0.83–0.98). (F) ROC curves comparing DMS-constrained circRNA structure models to published literature structures for select IRESes including Enterovirus A71, with individual AUC values indicated.

bioRxiv preprint doi: https://doi.org/10.64898/2026.05.19.726202; this version posted May 20, 2026. The copyright holder for this preprint (which was not certified by peer review) is the author/funder, who has granted bioRxiv a license to display the preprint in perpetuity. It is made available under a CC-BY 4.0 International license.

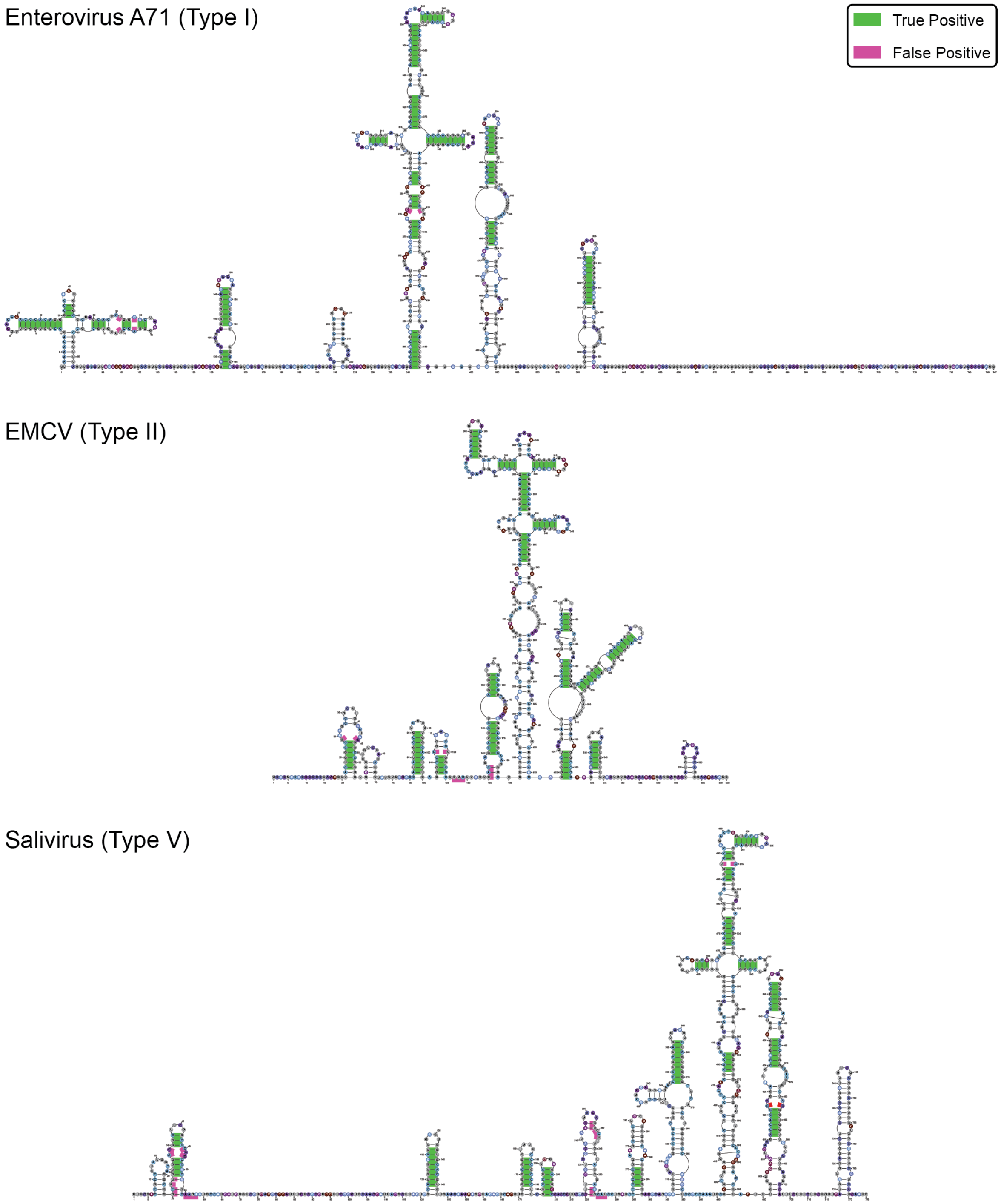

Figure S8: **Albatross-predicted base pairs overlaid on consensus literature structures.** Representative IRES secondary structures for Enterovirus A71 (Type I), EMCV (Type II), and Salivirus (Type V) are shown. Base pairs predicted by Albatross that agree with the consensus literature structures are colored as true positives (green), while Albatross predictions absent from the consensus literature structures are colored as false positives (pink).

bioRxiv preprint doi: https://doi.org/10.64898/2026.05.19.726202; this version posted May 20, 2026. The copyright holder for this preprint (which was not certified by peer review) is the author/funder, who has granted bioRxiv a license to display the preprint in perpetuity. It is made available under a CC-BY 4.0 International license.

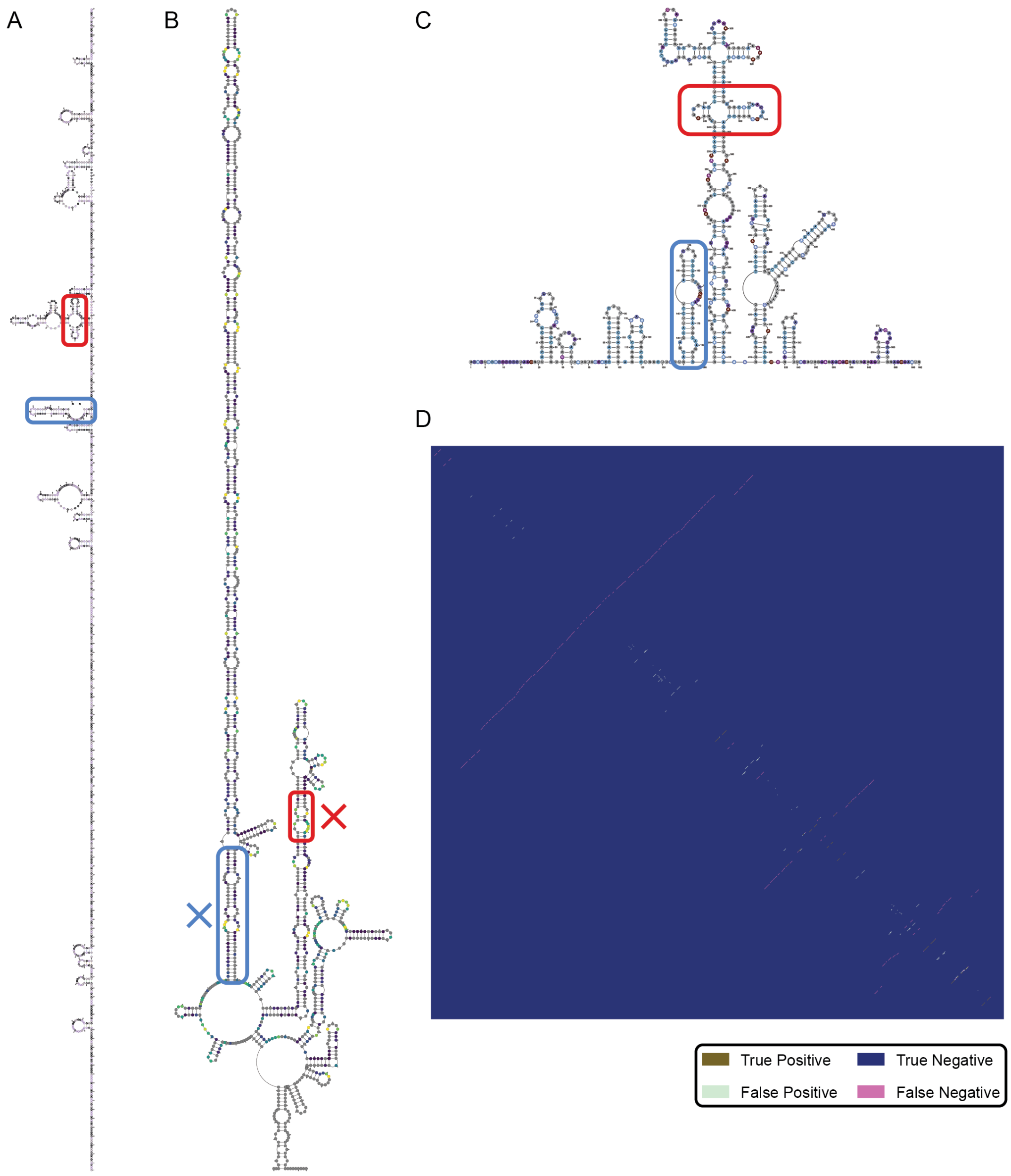

Figure S9: **Albatross correctly predicts Cosavirus A1 structure where DMS-constrained modeling creates a false stem.** (A) Albatross-predicted secondary structure, with red boxes highlighting stems scored as false positives against the DMS-constrained reference. (B) DMS-MaPseq constrained structure model, which incorrectly predicts an extended stem in place of the branched architecture. (C) Previously published Cosavirus A1 IRES secondary structure, confirming that the Albatross predictions in (A) are correct. (D) Full secondary structure with all Albatross predictions classified as true positives (green), true negatives (dark), false positives (red), and false negatives (marked with X). These cases demonstrate that Albatross true precision is higher than reported metrics indicate, and that benchmarking is limited by imperfections in the DMS-constrained reference structures.

bioRxiv preprint doi: https://doi.org/10.64898/2026.05.19.726202; this version posted May 20, 2026. The copyright holder for this preprint (which was not certified by peer review) is the author/funder, who has granted bioRxiv a license to display the preprint in perpetuity. It is made available under a CC-BY 4.0 International license.

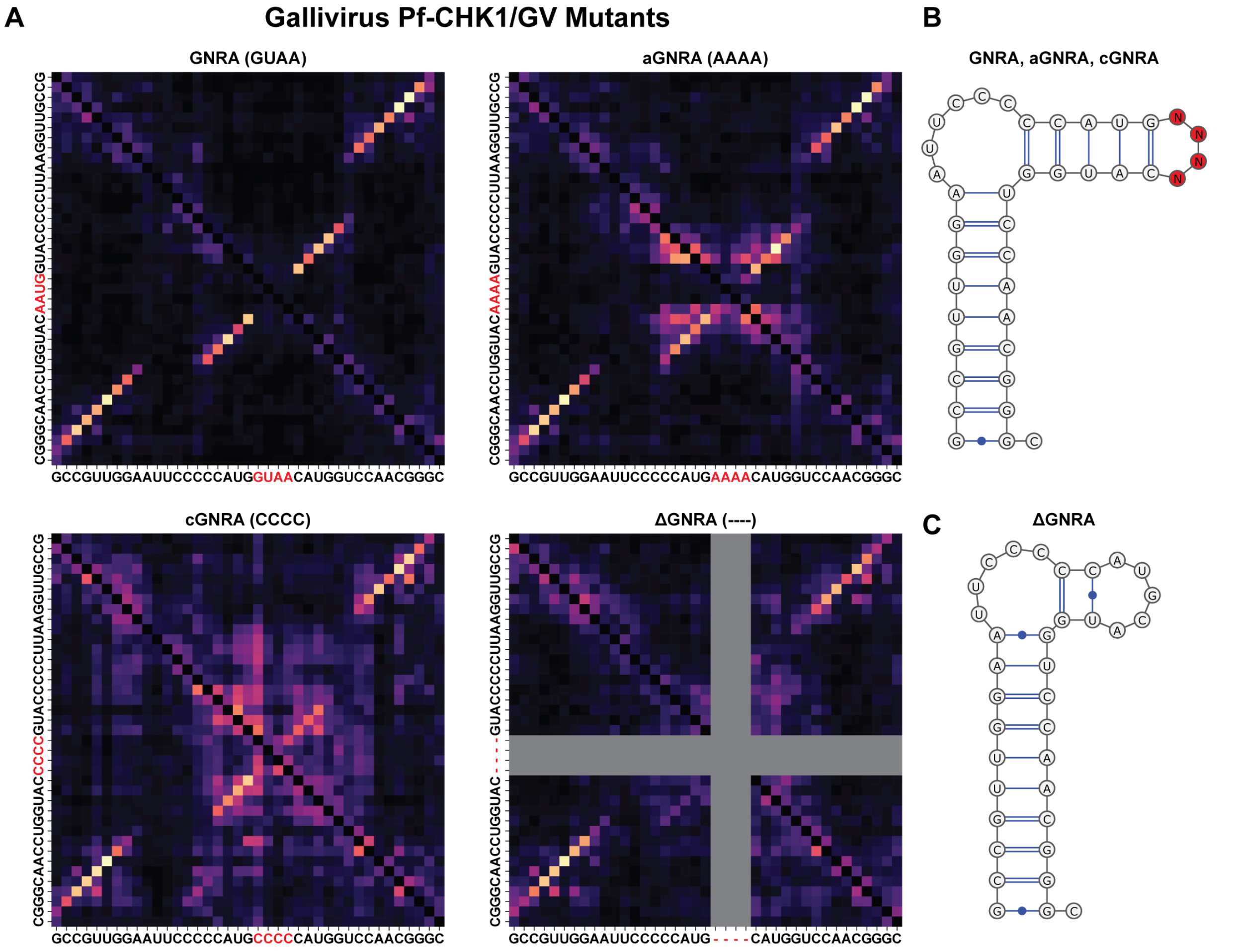

Figure S10: **Albatrossuses motif recognition to predict structure.** (**A**) Dependency maps of the Gallivirus IRES with GNRA mutations: wild-type (GUAA), AAAA, CCCC, and deletion. (**B,C**) Predicted structures for GNRA variants and deletion mutant.

bioRxiv preprint doi: https://doi.org/10.64898/2026.05.19.726202; this version posted May 20, 2026. The copyright holder for this preprint (which was not certified by peer review) is the author/funder, who has granted bioRxiv a license to display the preprint in perpetuity. It is made available under a CC-BY 4.0 International license.

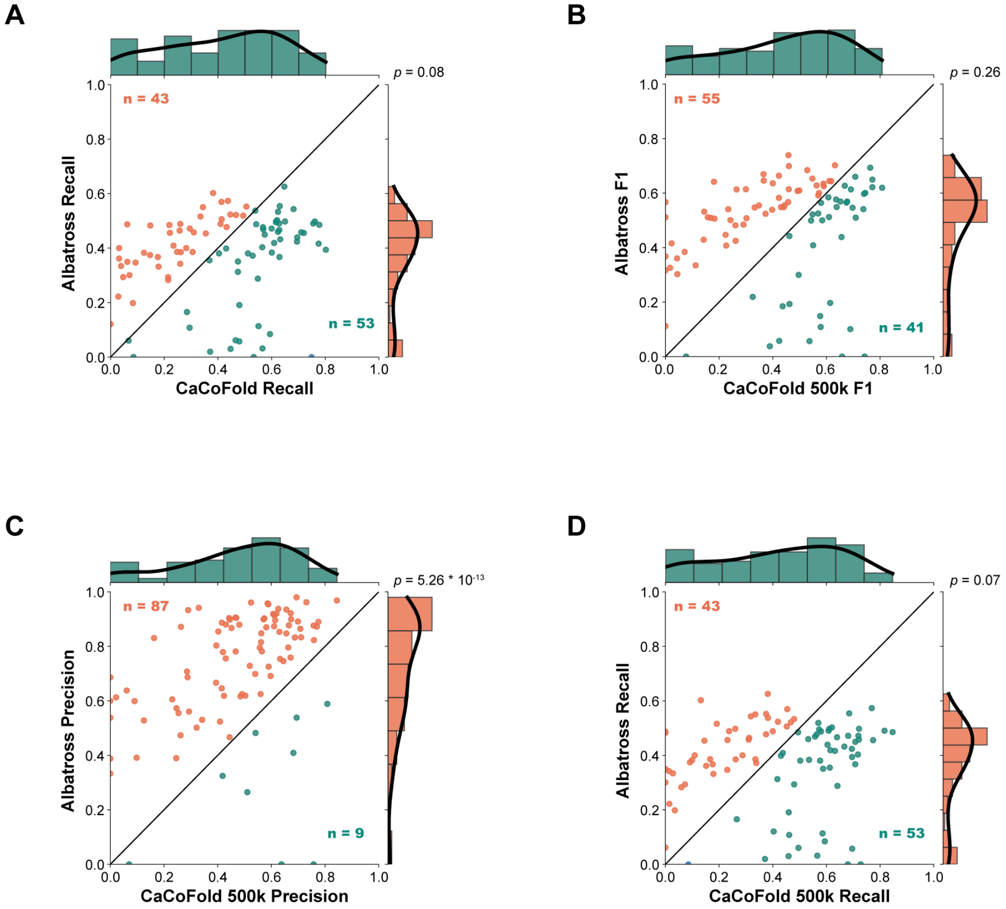

Figure S11: **Albatross outperforms covariation analysis** (**A**) Recall for Albatross versus CaCoFold across 96 IRESes *p*-values from a Mann-Whitney U test (n = 96). (**B, C, D**) F1, precision, and recall, respectively, across 96 IRESes for Albatross versus CaCoFold, where CaCoFold had access to the 500,000 sequences in the 500k training set. The 50k Albatross model was used. *p*-values from a Mann-Whitney U test (n = 96).

bioRxiv preprint doi: https://doi.org/10.64898/2026.05.19.726202; this version posted May 20, 2026. The copyright holder for this preprint (which was not certified by peer review) is the author/funder, who has granted bioRxiv a license to display the preprint in perpetuity. It is made available under a CC-BY 4.0 International license.

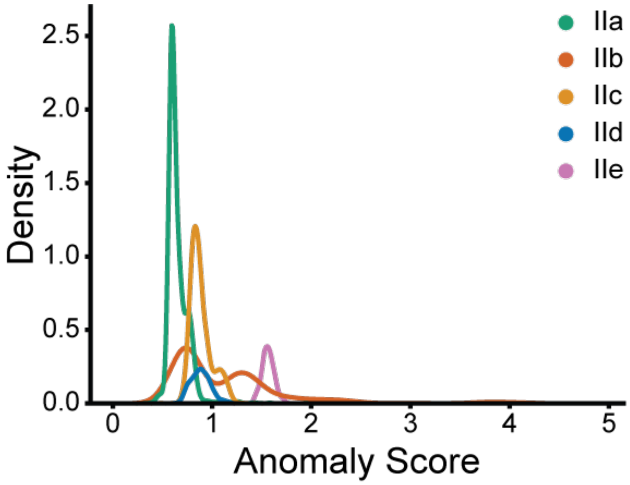

Figure S12: **Distribution of anomaly scores in spectral analysis clusters.** Kernel Density estimate of anomaly score distributions for each cluster from Figure 5B.

Table S1: **Albatross structure prediction performance across training sets.** Summary statistics corresponding to Figure 3E, F. F1 and precision (mean and median) are reported for each model across all 96 DMS-MaPseq constrained IRES structures. The pretrained model (no fine-tuning) shows near-zero performance. Fine-tuning on 200k and 500k sequence sets yields comparable F1 scores, while the smaller, diversity-balanced 50k training set achieves the highest F1 (median 0.54) with comparable precision (median 0.78). Precision remains consistently high across all fine-tuned models, reflecting the tendency of Albatross to predict base pairs that are nearly always correct.

Model	F1 Mean	F1 Median	Precision Mean	Precision Median
pretrained	0.05	0.04	0.10	0.07
200k	0.44	0.50	0.71	0.81
500k	0.45	0.49	0.68	0.76
50k	0.48	0.54	0.72	0.78

bioRxiv preprint doi: https://doi.org/10.64898/2026.05.19.726202; this version posted May 20, 2026. The copyright holder for this preprint (which was not certified by peer review) is the author/funder, who has granted bioRxiv a license to display the preprint in perpetuity. It is made available under a CC-BY 4.0 International license.

Table S2: **Albatross outperforms RScape and CaCoFold in F1 and precision.** Summary statistics corresponding to Figure 4E, F. F1 and precision (mean and median) are reported for each method (Albatross, RScape, CaCoFold) across all 96 DMS-MaPseq-constrained IRES structures. RScape and CaCoFold were compared being given either the 50k or 500k dataset for analysis. Bolded text indicates the highest value for that column.

Method	F1 Mean	F1 Median	Precision Mean	Precision Median	Recall Mean	Recall Median
Albatross	**0.48**	**0.54**	**0.72**	**0.78**	0.38	0.41
RScape 50k	0.01	0.00	0.30	0.00	0.01	0.00
RScape 500k	0.01	0.00	0.20	0.00	0.00	0.00
CaCoFold 50k	0.43	0.46	0.46	0.51	0.42	0.45
CaCoFold 500k	0.44	0.46	0.47	0.52	**0.42**	**0.46**

bioRxiv preprint doi: https://doi.org/10.64898/2026.05.19.726202; this version posted May 20, 2026. The copyright holder for this preprint (which was not certified by peer review) is the author/funder, who has granted bioRxiv a license to display the preprint in perpetuity. It is made available under a CC-BY 4.0 International license.

Caption for Data S1. **Detailed data for analysis of experiments.** An .xlsx file with 9 sheets.

1. "circRNA_qPCR": Corresponds to Figure S1. Sample indicates cDNA from either circular or linear forms of the IRES reporter library. Fluor indicates which fluorophore, namely JOE for the amplicon spanning oAS153 and FAM for the amplicon spanning oAS151. Rep is an indicator or separate replicates.
2. "IRESes": Corresponds to the distribution and sequences of the IRES reporter library. ID is an ID used to shorthand indicate the relevant IRES through analysis. Name is a name of the IRES source virus. Sequence with Barcode has the IRES sequence with the barcode sequence used to quantify presence and flanking primer binding sequences. IRES Sequence is just the sequence of the IRES. Barcode is the sequence of the barcode. Host indicates the canonical host of the virus. Type indicates the IRES type.
3. "Plasmid Library" corresponds to the data in Figure S2A, C, D. Reference indicates the IRES.
4. "circRNA Library" corresponds to the data in Figure S2B, C, D. Reference indicates the IRES.
5. "PolysomeProfiles" corresponds to the data in Figure S3. Cell indicates cell type. BioRep is an indicator for each biological replicate for that cell line. TechRep is an indicator for each technical replicate for that cell line. Position indicates the position of the plunger in millimeters. Abs260 and Abs280 indicate absorbance in arbitrary units at 260 nm and 280 nm respectively.
6. "Ribosomal_Loading" corresponds the the multi-cell IRES activity atlas (Figure 2C). ID indicates IRES, Ribosomal_Loading is the average ribosomes/RNA, Ribosomal_Loading_Std is the standard deviation of previous column. Name indicate the virus from which the IRES originates. Cell is the cell type in which the IRES was tested. Sequence is the IRES sequence. Host indicates the broad category of viral host for matching IRES.
7. "TropismGini" data corresponding to Figure 2E. ID indicates the IRES, pVal the uncorrected p-value, Gini the gini index.
8. "DMS-AUCs" lists the AUC-ROC values for each IRES in either the linear or circular RNA format.
9. "DMS-Pearson" lists the Pearson R values for the DMS reactivity for each IRES. The columns indicates which samples are being compared.
